# Genome-Wide Analysis of the NADK Gene Family in Plants

**DOI:** 10.1371/journal.pone.0101051

**Published:** 2014-06-26

**Authors:** Wen-Yan Li, Xiang Wang, Ri Li, Wen-Qiang Li, Kun-Ming Chen

**Affiliations:** State Key Laboratory of Crop Stress Biology in Arid Areas, College of Life Sciences, Northwest A&F University, Yangling, Shaanxi, China; National Institute of Plant Genome Research, India

## Abstract

**Background:**

NAD(H) kinase (NADK) is the key enzyme that catalyzes *de novo* synthesis of NADP(H) from NAD(H) for NADP(H)-based metabolic pathways. In plants, NADKs form functional subfamilies. Studies of these families in *Arabidopsis thaliana* indicate that they have undergone considerable evolutionary selection; however, the detailed evolutionary history and functions of the various NADKs in plants are not clearly understood.

**Principal Findings:**

We performed a comparative genomic analysis that identified 74 NADK gene homologs from 24 species representing the eight major plant lineages within the supergroup Plantae: glaucophytes, rhodophytes, chlorophytes, bryophytes, lycophytes, gymnosperms, monocots and eudicots. Phylogenetic and structural analysis classified these NADK genes into four well-conserved subfamilies with considerable variety in the domain organization and gene structure among subfamily members. In addition to the typical NAD_kinase domain, additional domains, such as adenylate kinase, dual-specificity phosphatase, and protein tyrosine phosphatase catalytic domains, were found in subfamily II. Interestingly, NADKs in subfamily III exhibited low sequence similarity (∼30%) in the kinase domain within the subfamily and with the other subfamilies. These observations suggest that gene fusion and exon shuffling may have occurred after gene duplication, leading to specific domain organization seen in subfamilies II and III, respectively. Further analysis of the exon/intron structures showed that single intron loss and gain had occurred, yielding the diversified gene structures, during the process of structural evolution of NADK family genes. Finally, both available global microarray data analysis and qRT-RCR experiments revealed that the NADK genes in *Arabidopsis* and *Oryza sativa* show different expression patterns in different developmental stages and under several different abiotic/biotic stresses and hormone treatments, underscoring the functional diversity and functional divergence of the NADK family in plants.

**Conclusions:**

These findings will facilitate further studies of the NADK family and provide valuable information for functional validation of this family in plants.

## Introduction

NAD(H) and NADP(H) are crucial coenzymes and play important and distinguishable roles in all living organisms [1]. NAD(H) is primarily involved in catabolic reactions, whereas NADP(H) participates in anabolic reactions, such as NADP(H)-dependent reductive anabolic pathways, signal transduction, and defense against oxidative stress [1], [2]. Hence, regulation of the intracellular balance of NAD(H) and NADP(H) is critical. NAD(H) kinase (NADK) is the key enzyme in the *de novo* biosynthesis of NADP(H), catalyzing the transfer of a phosphoryl group from ATP to NAD(H), and thus plays an important role in the regulation of intracellular NAD(H)/NADP(H) balance for NADP(H)-based metabolic pathways.

NADK genes have been found in nearly all living organisms, including Archaea, eubacteria and eukaryotes, except for the intracellular parasite *Chlamydia trachomatis* [3]. NADK genes have been cloned from a wide variety of species, including Archaea (*Methanococcus jannaschii* [4]), eubacteria (*Mycobacterium tuberculosis* [5]), *Escherichia coli* [6], yeast (*Saccharomyces cerevisiae* [7], [8]), humans (*Homo sapiens* [9]) and plants (*Arabidopsis thaliana* [10], [11], [12]). Moreover, the number of NADKs in different organisms varies, with most prokaryotic organisms, such as Archaea and eubacteria, having only one NADK, whereas most eukaryotic organisms, such as yeast and plants, have several NADKs. For example, *Euglena gracilis* [13] has two NADKs and *S. cerevisiae* [14], [15] and *Arabidopsis* have three [10], [11], [16].

The enzymatic properties of natural or recombinant NADKs from several organisms, including their substrate specificity and structural properties, especially of the active site, have been well characterized [2], [9], [17], [18], [19]. All characterized NADKs are homomultimers and exhibit species-dependent phosphoryl donor and acceptor specificity. Hence, these enzymes are classified into two groups: ATP-NAD kinases and inorganic polyphosphate poly(P)/ATP-NAD kinases, based on the phosphoryl donor specificity [2]. For example, NADKs from gram-positive bacteria (*M. tuberculosis*) and Archaea (*M. jannaschii*) utilize both ATP and poly(P) as phosphoryl donors and they phosphorylate NAD^+^/NADH, whereas NADKs from gram-negative bacteria (*E. coli*) and eukaryotes (*S. cerevisiae*, *Arabidopsis* and human) utilize ATP but not poly(P), and phosphorylate NAD^+^ and NAD^+^/NADH, respectively [2]. In addition, analysis of the sequences and crystal structures of NADKs identified three highly conserved and functionally important motifs within the NADK family (a GGDG motif, an NE/D motif and a Gly-rich motif) and a possible phosphate transfer mechanism [20], [21], [22], [23], [24].

The biological significance and physiological functions of NADKs have been characterized from studies of several organisms. For example, mutation of the single NADK gene in *M. tuberculosis* and *Salmonella enterica* is lethal [3], [4]. Similarly, mutation of all three NADK genes in *S. cerevisiae* (*utr1/yef1/pos5*) or two of the three genes (*utr1/pos5*) also causes lethality, whereas the respective NADK gene mutations are not lethal in *Arabidopsis* [11], [16], [25], [26]. In addition, studies of the physiological functions of NADKs in *S. cerevisiae* and humans revealed that NADKs play a major role in protecting living cells against oxidative stress [8], [15], [26], [27] because NADPH is vital in the intracellular anti-oxidative defense system of most organisms [28].

In plants, NADKs are involved in regulating redox balance, biotic and abiotic stress responses and various developmental processes. Notably, NADK was the first protein in plants demonstrated to be regulated by the calcium-sensing protein calmodulin (CaM) [29], and plant NADKs are divided into CaM-independent and CaM-regulated isoforms. CaM-dependent NADK is essential for survival of plants under difficult conditions and for protecting plants against invading pathogens by helping to provide reductants for the NADPH-dependent oxidative burst [29], [30], [31]. For example, CaM-dependent NADK activity, but not CaM-independent NADK activity, increases under cold stress in green bean [32], and decreased in response to high salinity and drought in tomato [33] and wheat [34]. In *Arabidopsis*, three genes encoding NADK (*NADK1/NADK2/NADK3*) have been identified and their physiological functions have been characterized [2], [35]. *AtNADK1* is located in the cytosol and expressed mainly in roots, *AtNADK2* is located in the chloroplasts and expressed mainly in leaves, whereas *AtNADK3* is located in the peroxisome and is strongly expressed in reproductive tissues, such as the stigma, pollen and carpel vasculature [10], [11], [36]. The *AtNADK1*-deficient mutant exhibits sensitivity to γ-irradiation and paraquat-induced oxidative stress [16]. The *AtNADK2*-deletion mutant displays hypersensitivity to environmental stresses that induce oxidative stress, such as UVB-irradiation, drought, heat shock and high salinity [11]. Similarly, the *AtNADK3*-null mutant shows hypersensitivity to oxidative stress, including methyl viologen, high salinity and osmotic shock [10]. Moreover, AtNADK2 plays a vital role in chlorophyll synthesis and protects chloroplasts against oxidative damage [11]. In addition, plants growth and fertility are affected in the NADK-deficient mutants [2], [11], [37].

Although these studies in bacteria, yeast, human and *Arabidopsis* have led to an understanding of the biochemical properties and physiological functions of NADKs, there has been no systematic study of the evolution and functional divergence of the NADK gene family, especially in Plantae. Here, we performed a comprehensive analysis of the NADK gene family in 24 species, representing the eight major plant lineages within the supergroup Plantae. Phylogenetic analysis was performed to delineate the evolutionary history of the NADK family in Plantae, and exon/intron structure analysis was performed to gain insight into the possible mechanisms of the structural diversity of NADK gene family. Finally, the tissue-specificity and inducibility of NADK gene expression in *Arabidopsis* and rice (*Oryza sativa*) were characterized by examining publicly available microarray datasets and by qRT-PCR experiments. The results obtained here will broaden our understanding of the roles of plant NADKs and provide a framework for further functional investigations of these genes in plants.

## Results

### Identification of NADK family members in plants

**Figure 1:**
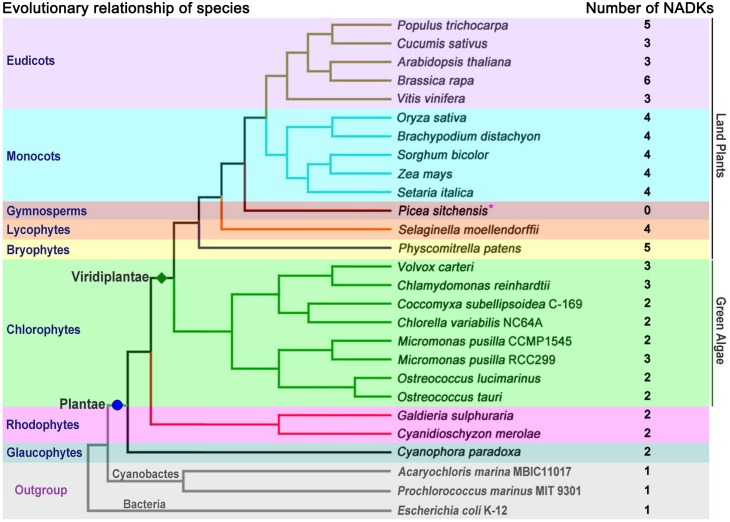
Systematic evolutionary relationships of 24 species among eight lineages within the supergroup Plantae. The numbers of NADK homologs in each species are listed next to the tree. *, the genome sequencing of *Picea sitchensis* is not complete.

To comprehensively investigate and characterize the NADK gene family in plants, 24 species representing the eight major plant lineages within the supergroup Plantae, were selected for analysis (Figure 1). A hidden Markov model (HMM) search was performed with the obtained sequences and 74 NADK homologs were identified (Figure 1, Table S1). Except for the gymnosperm *Picea sitchensis*, for which the full genome sequence was not yet available, two or more NADK genes were identified in each genome of the selected species (Figure 1). Most of the aquatic algae, including glaucophytes, rhodophytes and chlorophytes, contained two NADK genes per genome and only three species in the chlorophytes, *Micromonas pusilla* RCC299, *Chlamydomonas reinhardtii* and *Volvox carteri*, carried three NADK genes per genome (Figure 1). In contrast, three or more NADK genes were found in all land plants, including bryophytes, lycophytes, monocots and eudicots (Figure 1). In addition, only one NADK gene was identified per genome in the cyanophytes (*Acaryochloris marina* MBIC11017 and *Prochlorococcus marinus* MIT 9301) and bacteria (*E. coli* K-12), outgroup (Figure 1).

The Pfam and SMART databases were used to analysis the functional domains of the identified NADK candidates. All of the putative NADKs possess a typical NAD_kinase domain (Pfam accession number PF01513) and some also contain other functional domains such as an adenylate kinase domain (ADK; PF00406), a dual-specificity phosphatase, catalytic domain (DSPc; PF00782), and/or a protein tyrosine phosphatase, catalytic domain/DSPc domain (PTPc/DSPc; SMART accession number SM000012). Only one NADK candidate, CpNADK2, which did not contain a complete NAD_kinase domain, was excluded from the following analysis.

### Phylogenetic analysis and classification of the NADK family

**Figure 2:**
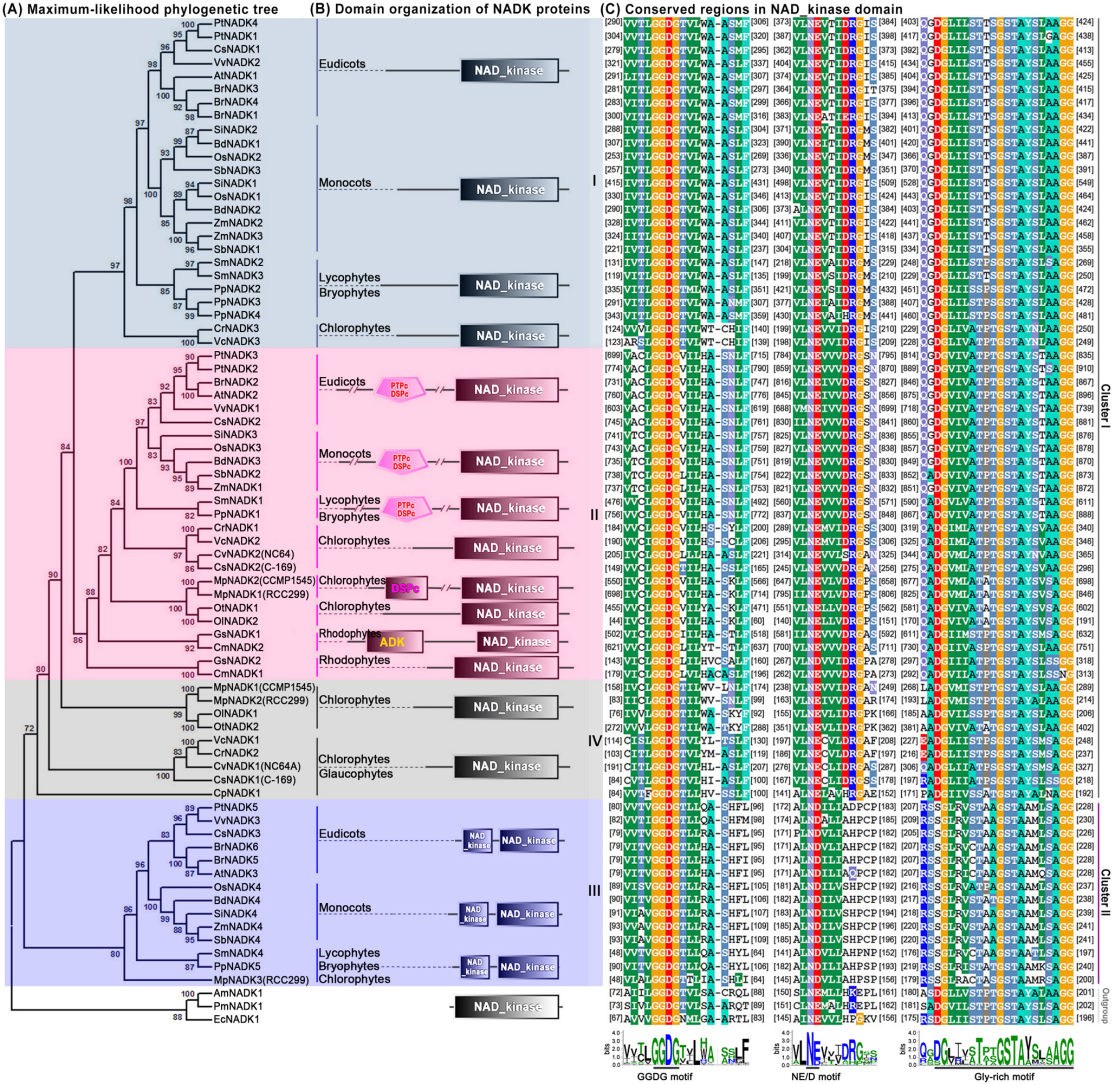
Phylogenetic relationships and domain organization of NADK genes in plants. (**A**) The rooted maximum-likelihood phylogenetic tree of NADK family members was inferred from the amino acid sequence alignment of the NAD_kinase domain. Numbers above the nodes represent bootstrap values from 1000 replications. (**B**) Domain organization of the NADKs. (**C**) Amino acid sequence alignment of conserved motifs within the NAD_kinase domain.

To explore the phylogenetic relationships among NADK family members in plants, we first generated a rooted maximum-likelihood phylogenetic tree with the 73 NADKs from the 24 species (Figure 2A), which was inferred from the amino acid sequences of their NAD_kinase domains (Figure S1). Furthermore, phylogenetic trees reconstructed by the neighbor joining, minimum evolution and maximum parsimony methods showed very similar topologies to the maximum-likelihood tree (data not shown). Using *EcNADK1*, *AmNADK1* and *PmNADK1* from bacteria and cyanophytes as the outgroup, all NADK homologs in plants can be classified into four well-conserved subfamilies (I–IV; Figure 2A) with high statistical support, according to the topology and the deep duplication nodes of NADK paralogues in the maximum-likelihood tree. Interestingly, the topological relationship of members within subfamilies was highly consistent with the evolutionary relationships between species in Plantae (Figures 1 and 2A). The majority of aquatic algae contained two NADK genes per genome and grouped into subfamilies II and IV, whereas the majority of land plants, except for the gymnosperm *Picea sitchensis*, carried ≥ three NADK genes per genome and were clustered into subfamilies I, II and III (Figure 2A). Moreover, only one of the NADK genes from green algae (*CrNADK3*) fell into subfamily I and only two (*VcNADK3* and *MpNADK3*) fell into subfamily III (Figure 2A).

Examination of the chromosomal locations of the NADK family genes in the genomes of algae (*Ostreococcus lucimarinus*, *Ostreococcus tauri*, *C. reinhardtii* and *V. carteri*), bryophytes (*Physcomitrella patens*), lycophytes (*Selaginella moellendorffii*), monocots (*Brachypodium distachyon*, rice, *Sorghum bicolor* and *Zea mays*) and eudicots (*Arabidopsis*, *Brassica rapa*, *Populus trichocarpa* and *Vitis vinifera*) showed that NADK genes are randomly distributed (Figure S3). Moreover, a search for NADK paralogs using the Plant Genome Duplication Database (PGDD; http://chibba.agtec.uga.edu/duplication/) [38] revealed eight paralogous gene pairs in *P. patens*, rice, *Z. mays*, *B. rapa* and *P. trichocarpa* (Figure S4A), but none in the other species.

To further explore association of positive selection with duplication and divergence of NADK family genes, the rate of non-synonymous substitution (Ka), synonymous substitution (Ks) and the Ka/Ks ratios were calculated for the eight paralogous gene pairs and used to estimate duplication and divergence times. The Ka/Ks ratios varied from 0.18 to 0.39 among the five different species (Figure S4B). The fact that the Ka/Ks ratios were <1 suggests that the NADK family genes have undergone strong negative selection pressure, and the duplication event was estimated to have occurred ∼13.8–67.6 million years ago. Divergence of *P. patens*, rice, *Z. mays*, *P. trichocarpa* and *B. rapa* was estimated to have occurred 67.6, 61.5, 13.8, 20.8 and 23.1–33.1 million years ago, respectively (Figure S4B).

Further analysis of the functional domains showed that domain organization of NADKs in the different subfamilies varied considerably (Figure 2B). All of the identified NADKs contained a typical NAD_kinase domain at the C terminus, but the proteins in subfamily II also carried the additional N-terminal catalytic domains noted earlier (DSPc, PTPc/DSPc or ADK; Figure 2B). Interestingly, the NAD_kinase domain of proteins in subfamily III was not only divided into two parts, but also exhibited low sequence similarity (∼30%) between subfamily III members and the other subfamilies (Figures 2C and S1). In addition, based on the NAD_kinase domain sequence similarity, the NADK gene subfamilies could be further divided into two clusters: cluster I (subfamilies I, II and IV) and cluster II (subfamily III).

More detailed analysis of the NAD_kinase domains of the 73 NADKs revealed three highly conserved and functionally important motifs: a GGDG motif, an NE/D motif and a Gly-rich motif (Figures 2C and S1). The GGDG motif is involved in ATP-binding, whereas the NE/D and Gly-rich motifs are involved in NAD(H) binding [2], [20], [21], [23], [24].

### Structure analysis of NADK family genes

**Figure 3:**
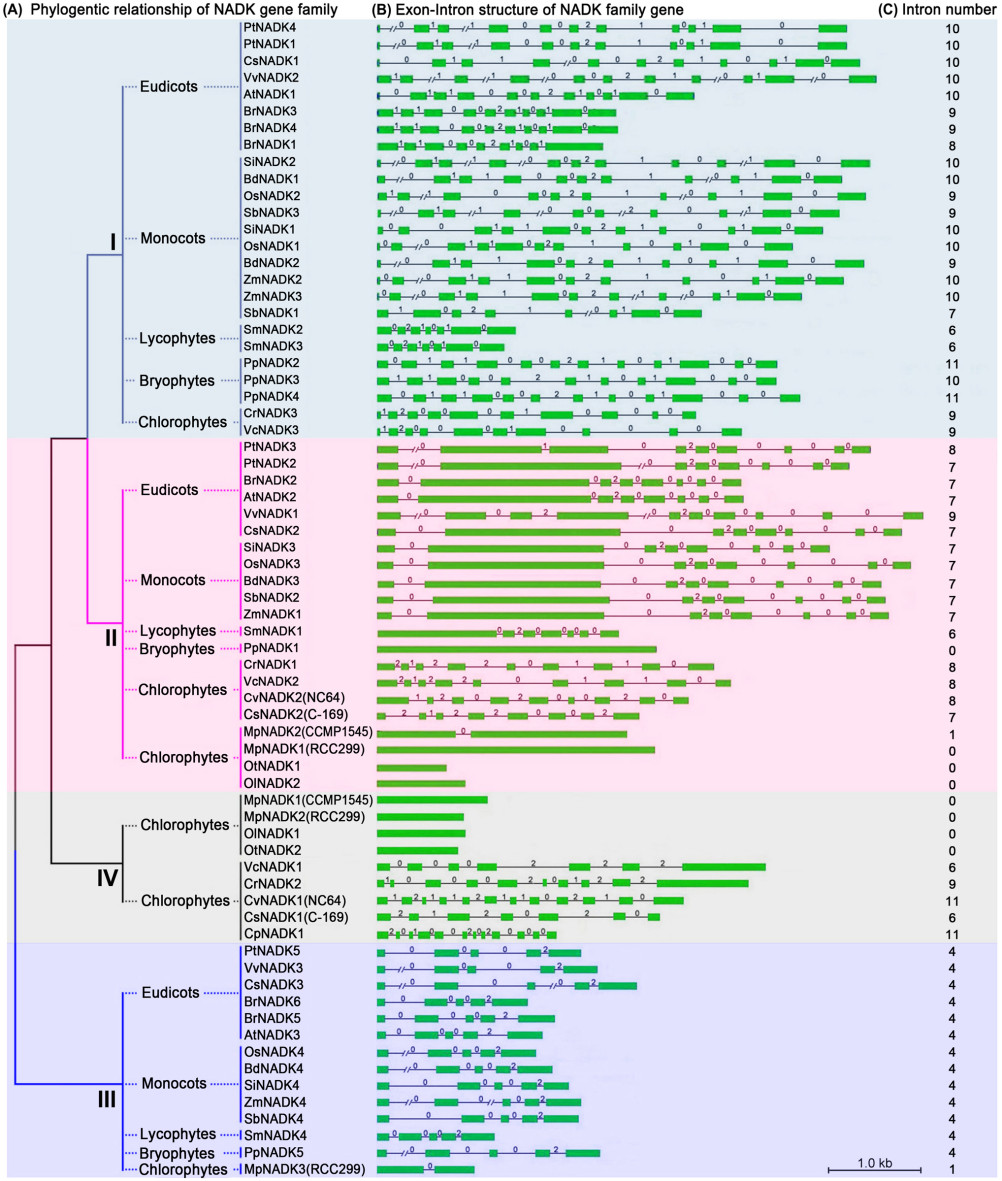
Exon/intron structure of NADK family genes of green plants. Green boxes represent exons; black lines represent introns; numbers 0, 1 and 2 are intron phases. The length of the boxes and lines are scaled relative to the length of the gene, and longer introns are denoted by a double slash.

Intron position is generally very well-conserved in orthologous genes over long evolutionary time intervals, whereas exon/intron structure is slightly less, but sufficiently, conserved in paralogous genes to reveal evolutionary relationships between introns [39], [40], [41]. To investigate the gene structural diversity and possible mechanisms for the structural evolution of NADK homologs in green plants (Viridiplantae, excluding glaucophytes and rhodophytes), we analyzed the exon/intron organization in the coding sequence. Overall, there was considerable diversity in the number of introns (0–11) and the length of introns (50–7452 bp) in the NADK family genes (Figure 3). Interestingly, NADK family members within the same subfamily shared similar gene structure in terms of intron number, exon length, and/or intron phases, with the exception of subfamily IV (Figure 3). For instance, NADK genes in subfamily I had 6–11 introns, 48% (12/25) and 84% (21/25) of which contained 10 and 9–11 introns, respectively. We also investigated intron phases with respect to codons in the NADK genes. The intron phases were remarkably well conserved among subfamily members, whereas the intron arrangement and phases were strikingly distinct between subfamilies (Figure 3B).

**Figure 4:**
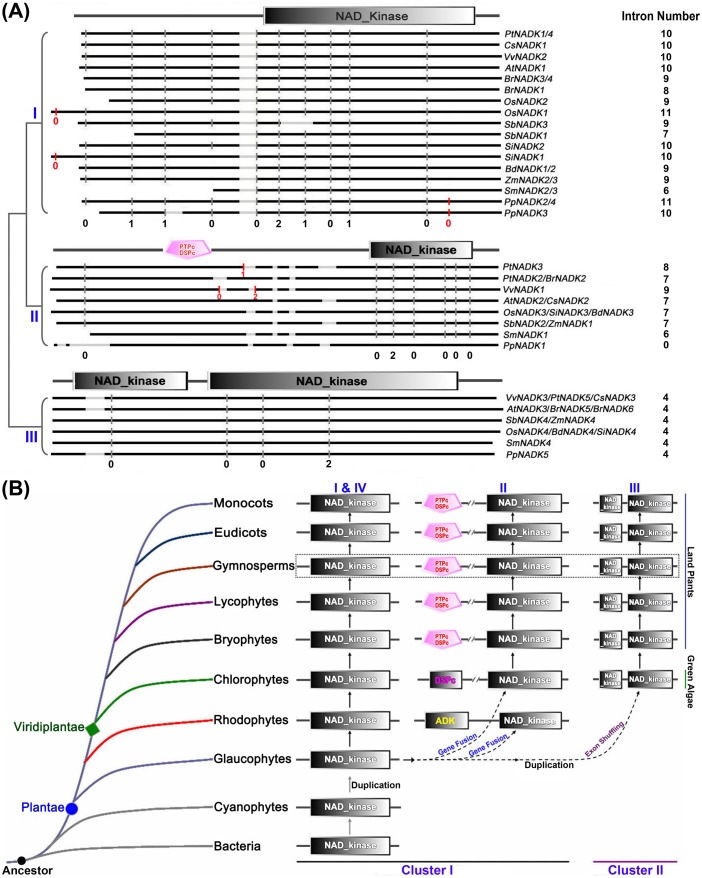
The expansion and evolution of the NADK gene family in plants. (**A**) Schematic comparison of intron distribution in NADK orthologs of land plants generated with the CIWOG software. Black horizontal lines are aligned sequences; gray horizontal lines are gaps in the alignment; gray vertical bars are conserved common introns; red vertical bars are gained introns. The numbers 0, 1 and 2 are intron phases. (**B**) A model for the expansion and evolution of the NADK gene family in Plantae.

To further explore intron loss or gain within the NADK family genes, we next examined the exon/intron organization of paralogs and orthologs in the land plants (Figure 4A). This analysis revealed that single intron loss and gain likely occurred during the structural evolution of NADK family genes in land plants. For example, The paralogous genes *PtNADK1/4* and *SmNADK2/3* showed conserved exon/intron structure in terms of the number of introns and exon length, whereas a single intron appears to have been lost during the evolution of the *BrNADK1/4* and *PpNADK3/4* paralogs (Figures 3B and 4A). By contrast, a single intron gain occurred between the paralogs *PtNADK2* and *3* (Figures 3B and 4A). Among the orthologous NADK genes in land plants, the majority of subfamilies I, II and III members contained 10, 7 and 4 conserved common introns, respectively (Figure 4A), whereas some subfamily I and II NADK orthologs contained fewer introns. For example, *OsNADK1*, *SiNADK1*, *BdNADK1/2*, *ZmNADK2/3* (subfamily I) have only nine conserved common introns. Similar intron loses were seen with *BrNADK1/3/4*, *SbNADK3* and *SmNADK2/3* (subfamily I), *PpNADK1* (subfamily II), and *SmNADK4* (subfamily III; Figure 4A). Single intron gain was also observed in the NADK genes of land plants. For instance, *OsNADK1*, *SiNADK1*, and *PpNADK2/3/4* in subfamily I and *PtNADK3* in subfamily II appear to have gained an intron, whereas *VvNADK1* in subfamily II appears to have gained two introns (Figure 4A).

### Tissue-specific expression patterns of NADK genes in *Arabidopsis* and rice

**Figure 5:**
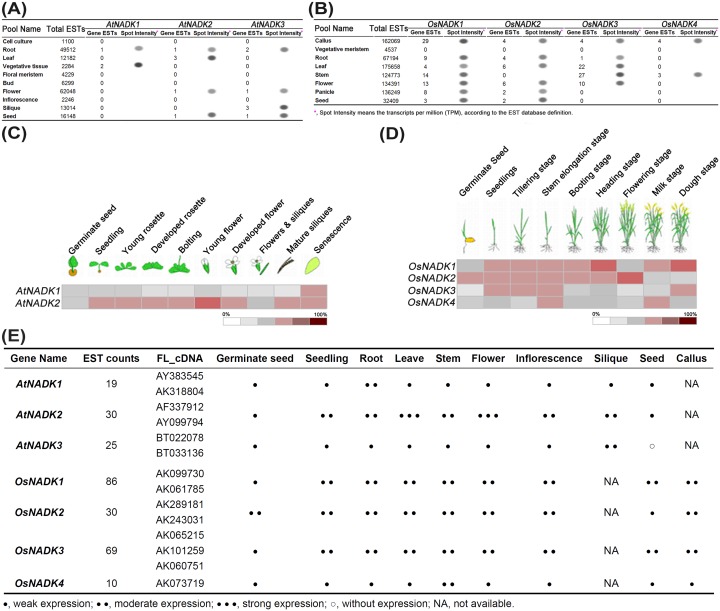
Developmental expression patterns of NADK family genes in *Arabidopsis* and rice. Expression profiles of (**A**) *AtNADKs* and (**B**) *OsNADKs* inferred from public EST data. Expression profiles of (**C**) *AtNADKs* (except *AtNADK3*) and (**D**) *OsNADKs* in different developmental stages obtained from microarray data reported in Genevestigator. Results are shown as heat maps in white/gray/red (low to high) that reflect the percent of expression. (**E**) Summary of the relative expression patterns of AtNADK and OsNADK genes inferred from the combined EST (A, B; Tables S3 and S4) and microarray (C, D) profiles.

To investigate differences in expression of NADK genes in *Arabidopsis* and rice during plant development, we first analyzed the expression profiles of AtNADK and OsNADK genes available in expressed sequence tag (EST) databases from vegetative and reproductive development stages (Figures 5A and 5B and Tables S3 and S4, respectively). Varying levels of ESTs for each of the AtNADK and OsNADK genes were found the database, indicating that all of the NADK genes in *Arabidopsis* and rice are expressed, and that they are likely differential expressed in different tissues or developmental stages. We then used *Arabidopsis* (ATH1, 22 k array) and rice (Os 51 k array) microarray data in Genevestigator to analyze the expression patterns of the NADK genes in 10 and 9 developmental stages/tissues, respectively (Figure 5C, D and S5). Essentially identical developmental expression profiles for the AtNADK and OsNADK genes were also obtained with data from the *Arabidopsis* and rice eFP browsers in the Bio-Analytic Resource (http://bar.utoronto.ca/welcome.htm) database [42]. *AtNADK3* was not included in the comparison of tissue/developmental expression patterns because it was not included in the microarray data. The combined expression patterns of *AtNADKs* and *OsNADKs* from the EST and microarray data analyses are summarized in Figure 5E.

Overall, all of the AtNADK and OsNADK genes were expressed during the vegetative and reproductive development stages, and they displayed strong tissue specificity. In *Arabidopsis*, *AtNADK2* showed higher expression in most tissues and developmental stages than *AtNADK1* (Figures 5C and S5). The highest *AtNADK2* expression is seen in leaves and flowers (Figures 5A, C and S5), consistent with Waller et al. (2010) [36]; whereas *AtNADK1* expression is highest in the dry seed and senescence stages (Figures 5C and S5). *AtNADK3* appears to be mainly expressed in the reproductive tissues such as flowers and siliques (Figure 5A), also consistent with the findings of Waller et al. (2010) [36]. In rice, the *OsNADK1*, *OsNADK2* and *OsNADK3* transcripts tend to accumulate to higher levels than the *OsNADK4* transcript in most tissues/developmental stages (Figures 5B, D and S5), but their individual expression patterns differ. For instance, during the germinate seed stage, *OsNADK1* transcript levels are low compared to *OsNADK2* (Figure 5D), whereas the opposite is observed in callus (Figure 5B). The highest levels of *OsNADK3* transcript are seen in leaf and stem (Figures 5D and S5); whereas relatively low levels of the Os*NADK4* transcript are seen in most tissues/developmental stages except for stem and inflorescence (Figures 5D and S5).

### Response profiles of NADK genes under abiotic/biotic stresses and hormone treatments in *Arabidopsis* and rice

To understand the molecular mechanism of *AtNADKs* and *OsNADKs* transcriptional regulation under abiotic/biotic stresses and hormone treatments, we first identified potential *cis*-elements in the promoter regions of each NADK gene using the PlantCARE program (Tables 1 and S2). We found that the promoter regions of AtNADK and OsNADK genes contain response elements for several abiotic/biotic stresses, such as low temperature, heat, drought (MYB binding sites), anaerobic conditions, and pathogens (TC-rich repeats, W box, GCC box, Box S, Box-W1 and EIRE) (Tables 1 and S2). In addition, the AtNADK and OsNADK gene promoters contain *cis*-elements for responding to several hormones, such as auxin, gibberellin, abscisic acid (ABA), ethylene, salicylic acid (SA) and methyl jasmonic acid (MeJA) (Tables 1 and S2). Further analysis showed that all of the *AtNADK* and *OsNADK* promoters contain anaerobic- and ABA-responsive elements; and the majority of them also carry heat stress, drought (MYB binding sites) and MeJA response elements (Tables 1 and S2).

**Table 1:** Abiotic/biotic stress and hormone response elements in *AtNADK* and *OsNADK* promoters*.

Cis-elements	Core sequences	Functions of the cis-elements	*OsNADK1*	*OsNADK2*	*OsNADK3*	*OsNADK4*	*AtNADK1*	*AtNADK2*	*AtNADK3*
**Abiotic/biotic stress**									
LTR	CCGAAA	low-temperature responsiveness		**√**		**√**			
HSE	AAAAAATTTC	heat-stress responsiveness	**√**	**√**	**√**	**√**	**√**		**√**
MBS	TAACTG	MYB binding site,drought inducibility	**√**	**√**	**√**	**√**			**√**
ARE	TGGTTT	anaerobic induction	**√**	**√**	**√**	**√**	**√**	**√**	**√**
TC-rich repeats	ATTCTCTAAC	defense and stressresponsiveness		**√**	**√**			**√**	**√**
W box	TTGACC	wound and pathogenresponsiveness			**√**		**√**		
Box S	AGCCACC	wound and pathogenresponsiveness	**√**						
GCC box	AGCCGCC	wound and pathogenresponsiveness				**√**			
EIRE	TTCGACC	Elicitor responsiveness						**√**	
Box-W1	TTGACC	fungal elicitorresponsiveness			**√**		**√**		**√**
**Hormone response elements**									
AuxRR-core	GGTCCAT	auxin responsiveness			**√**				
TGA-element	AACGAC	Auxin responsiveness					**√**	**√**	
GARE-motif	AAACAGA	Gibberellin responsiveness		**√**			**√**		
ABRE	CACGTG	abscisic acid responsiveness	**√**	**√**	**√**	**√**	**√**	**√**	**√**
ERE	ATTTCAAA	Ethylene responsiveness	**√**	**√**	**√**		**√**		
TCA-element	GAGAAGAATA	salicylic acid responsiveness					**√**		**√**
CGTCA-motif	CGTCA	MeJA responsiveness	**√**			**√**	**√**	**√**	**√**

*the *cis*-elements were identified with the PlantCARE program (http://bioinformatics.psb.ugent.be/webtools/plantcare/html/) using the sequences 1500 bp upstream from the transcription start site of each NADK gene. The “√” means the NADK gene contains this *cis*-element in the promoter region.

**Figure 6:**
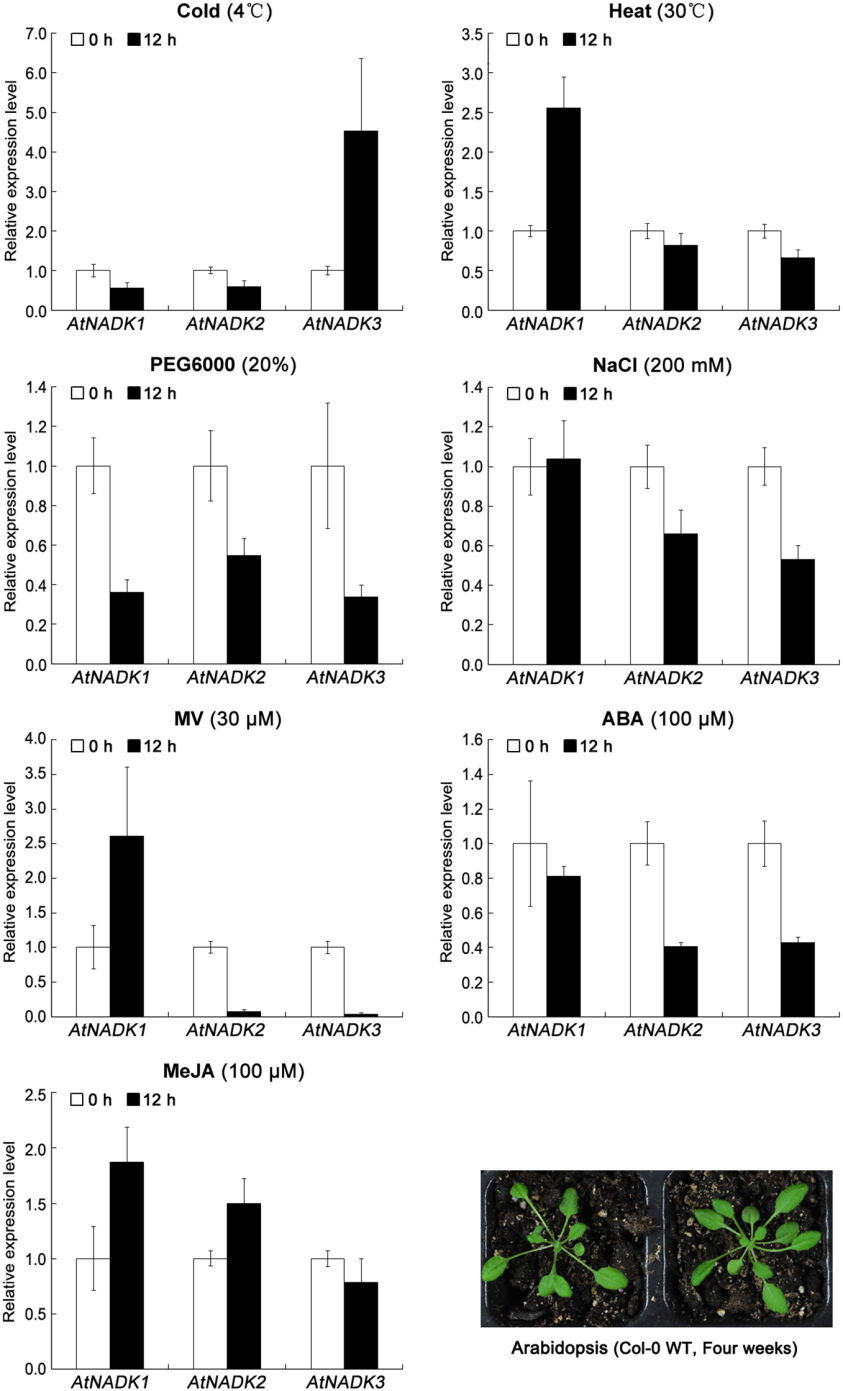
Expression patterns of NADK family genes in *Arabidopsis* under abiotic stress and hormone treatments. Expression levels of *AtNADK1–3* assayed by qRT-PCR under cold (4°C), heat (30°C), drought (20% PEG6000), salt (200 mM NaCl), oxidative (30 µM MV) stresses and MeJA (100 µM), ABA (100 µM) hormone treatments. Data are means ± SD (n = 3) and are representative of similar results from three independent experiments.

**Figure 7:**
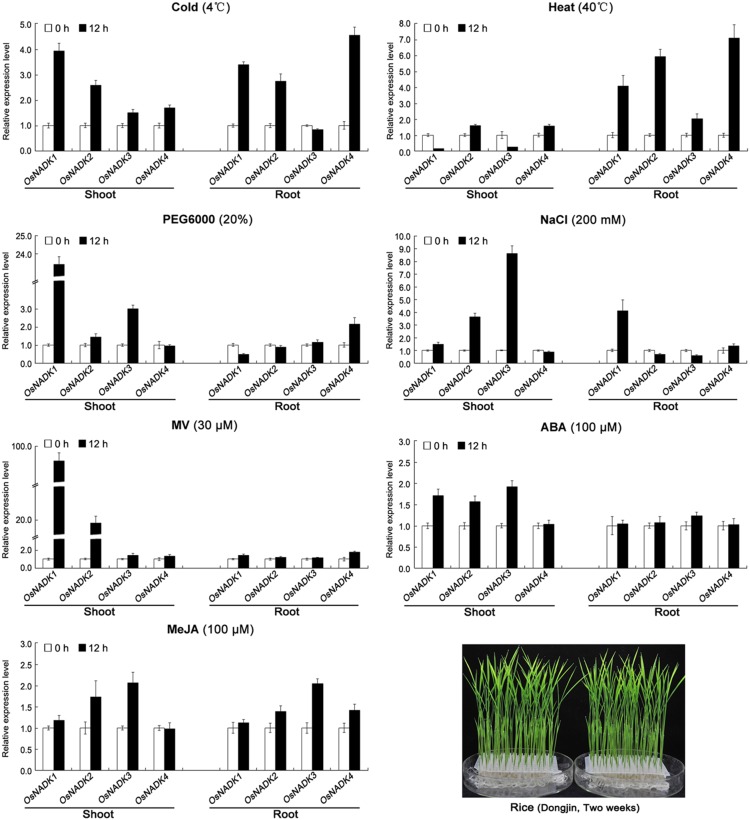
Expression patterns of NADK family genes in rice under abiotic stress and hormone treatments. Expression levels of *OsNADK1–4* assayed by qRT-PCR under cold (4°C), heat (30°C), drought (20% PEG6000), salt (200 mM NaCl), oxidative (30 µM MV) stresses and MeJA (100 µM), ABA (100 µM) hormone treatments. Data are means ± SD (n = 3) and are representative of similar results from three independent experiments.

To further demonstrate that the expression of AtNADK and OsNADK genes is induced by abiotic and biotic stress, we again examined *Arabidopsis* and rice microarray data in the Genevestigator database, as well as by qRT-PCR experiments. As shown in Figures 6, 7 and S6, the expression of AtNADK and OsNADK genes is induced to varying degrees by abiotic stress such as cold, heat, drought (PEG), salt (NaCl) and oxidative (methyl viologen, MV), as well as by biotic stress such as the pathogens *Botrytis cinerea*, *Blumeria graminis*, *Pseudomonas syringae*, *Agrobacterium tumefaciens*, *Mycosphaerella graminicola*, *Magnaporthe oryzae*, and *Magnaporthe grisea*. In *Arabidopsis*, *AtNADK1* is up-regulated in whole plants under heat and MV stresses, whereas it is down-regulated and does not change under PEG and cold treatment, respectively (Figure 6). By contrast, *AtNADK2* is down-regulated or does not change under these conditions (Figure 6). *AtNADK3* is up-regulated under cold stress, while it is down-regulated under other abiotic stresses including heat, PEG, NaCl, and MV (Figure 6). Additionally, both *AtNADK1* and *AtNADK2* can be up-regulated by some biotic stresses, although with different expression profiles under the same stress (Figure S6). In rice, *OsNADK1*/*2* are up-regulated in shoot and root under cold stress, in root under heat stress and in shoot under MV stress, respectively; while they are slightly down-regulated or does not change under these conditions (Figure 7). *OsNADK3* shows up-regulated under PEG in shoot and down-regulated or does not change under other abiotic stresses such as cold, heat, NaCl and MV (Figure 7). Similarly, *OsNADK4* is up-regulated in root under cold and heat and down-regulated or does not change under other abiotic stresses including PEG, NaCl, and MV (Figure 7). Additionally, *OsNADK1–4* can be induced by biotic stresses. For instance, *OsNADK1* is up-regulated in callus after infection by *A. tumefaciens* and root after infection by *M. oryzae*, whereas they are down-regulated in root and leaf after infection by *M. graminicola* and *M. grisea*, respectively (Figure S6).

As several hormone response elements were identified in the promoter regions of the AtNADK and OsNADK genes, we also examined their expression profiles under phytohormone treatments in the public *Arabidopsis* and rice microarray data (Figure S7), as well as by qRT-PCR experiments (Figures 6 and 7). *AtNADK1* and *AtNADK2* are differentially expressed in seedlings and cells treated with phytohormones, such as the auxins indole-3-acetic acid (IAA) and naphthaleneacetic acid (NAA), zeatin, SA, ABA, and ABA + SA (Figure S7). Interestingly, *AtNADK1* is up-regulated by ABA, ABA + SA, and ABA + MeJA treatment; whereas *AtNADK2* is down-regulation by ABA and ABA + SA treatment (Figure S7). The expressions of AtNADK genes by qRT-PCR showed *AtNADK1–2* are up-regulated by MeJA treatment and down-regulated or do not change by ABA treatment; *AtNADK3* is down-regulated by ABA treatment and does not change by MeJA treatment (Figure 6). In rice, *OsNADK1–4* are differentially expressed in seedlings and leaves treated with IAA, NAA, zeatin, gibberellin, kinetin, ABA, SA and jasmonic acid (JA) (Figure S7). For instance, *OsNADK3* is up-regulated in roots under *trans*-zeatin treatment, whereas *OsNADK4* is down-regulated in leaves under the same treatment (Figure S7). It is also notable that *OsNADK1–3* can be induced in shoot by ABA treatment and do not change in root, but *OsNADK4* is not obviously changed with the same treatment. All *OsNADK1*–*4* were up-regulated or not obviously changed by MeJA treatment.

## Discussion

### Gene fusion and exon shuffling after gene duplication contributed to the expansion and evolution of the NADK family in plants

Gene duplication is a common phenomenon in eukaryotes and contributes to biological diversity during evolution [43], [44], [45]. The NADKs are represented by at least one gene in nearly every living organism. In this study, we found that the 24 representative plant species from the supergroup Plantae examined contain 2–6 NADK homologs, whereas cyanophytes, considered to be the ancestor of Plantae, had only one NADK homolog per genome, suggesting that a single gene duplication leading to the expansion of the NADK gene family, occurred during the divergence of ancestral cyanophytes from Plantae. The scattered distribution of NADK family genes on chromosomes (Figure S3) and the eight paralogous NADK gene pairs found in five land plants (Figure S4) suggest that segmental duplications may have been involved in the expansion of the NADK gene family and caused differences in the number of NADK genes within subfamilies and species of land plants after divergence from aquatic algae.

Gene fusion and exon shuffling after gene duplication are mechanisms that can enhance the functional divergence of duplicated genes by creating additional domains or rearranging the original functional domains [46], [47], [48], [49]. In this study, phylogenetic analysis, together with the domain organizations and gene structures of NADK family genes, showed distinct evolutionary differences among subfamilies (Figure 2). Therefore, a model was constructed to account for the expansion and evolution of the NADK gene family in plants (Figure 4B). In this model, all NADK family members originated from a common ancestor, which contained only the typical NAD_kinase domain and existed in all living organisms from prokaryotic bacteria to eukaryotic angiosperms; cyanophytes were considered to be the ancestor of Plantae, and at least one gene duplication occurred to yield two NADK genes in eukaryotic glaucophytes (Figures 1 and 4B). Moreover, during the evolutionary diversification from glaucophytes to rhodophytes and Viridiplantae, gene fusion events occurred and additional catalytic domains (ADK, DSPc or PTPc/DSPc) were acquired in the N-terminus, leading to the diversified domain organization seen in subfamily II (Figures 2B and 4B). In addition, exon shuffling after gene duplication contributed to the formation of the bifurcated NAD_kinase domain in subfamily III (Figures 2B and 4B).

### Single intron loss and gain lead to the diversified gene structures

Gene structural diversity within gene families is another evolutionary mechanism that promotes variability, and intron loss or gain is important in generating structural diversity and complexity [41], [50]. Analyzing the exon/intron structures of NADK genes, we found that the number of introns and intron phases among subfamily members are remarkably conserved, whereas the intron arrangement and intron phases are strikingly distinct between subfamilies (Figures 3 and 4). Further analysis of the orthologous and paralogous genes in land plants (Figure 4) suggests that single intron loss as well as intron gain likely occurred and contributed to the diversification of gene structure, and consequent functional diversity and divergence, during the evolution of the NADK family in plants.

### NADKs are involved in plant responses to several abiotic and biotic stresses

The three NADK genes of *Arabidopsis* belong to three different subfamilies according to our phylogenetic analysis (Figures 1 and 2). The three *AtNADKs* are known to have anti-oxidative functions [10], [11], [12], [16], [36], but they have distinct mechanisms by which they facilitate plant resistance to oxidative stress. AtNADK1 (subfamily I) is a cytosolic enzyme that indirectly provides cytosolic NADP for plasma membrane NADPH oxidases, which are the key producers of reactive oxygen species under both normal and stress conditions in plants [36], [51], [52], [53]. AtNADK2 (subfamily II) is a chloroplastic enzyme that plays a vital role in chlorophyll synthesis and chloroplast protection against oxidative damage by regulating plastidic NADP-biosynthesis [11], [36]. AtNADK3 (subfamily III) is a peroxisomal enzyme that plays a prominent anti-oxidation role by providing the peroxisomal reductant NADPH [10], [36]. NADK genes in subfamily IV were only present in aquatic algae and grouped with *S. cerevisiae NADK1* (Pos5) in our broader phylogenetic analysis (Figure S2). Pos5 is located in the mitochondria and its deletion causes slow growth, sensitivity to oxidative stress, such as paraquat, hyperoxia, H_2_O_2_, and Cu^2+^, and biosynthesis deficiency on iron-sulfur clusters [25], [54], [55]. These observations suggest that plant NADKs in subfamily IV may also localize to mitochondria and protect against oxidative damage by regulating mitochondrial NADP-biosynthesis.

We found that plant NADKs are also involved in responding to several abiotic/biotic stresses, including cold, heat, drought, salt, oxidative and pathogens. *Arabidopsis NADK1* expression is up-regulated by H_2_O_2_, irradiation and the bacterial pathogen *P. syringae* pv. *tomato*; and the *AtNADK1* deficient mutant is sensitive to γ-irradiation and paraquat-induced oxidative stress [16]. The *AtNADK2* deletion mutant is also hypersensitive to environmental stresses that trigger oxidative stress, such as UVB-irradiation, drought, heat, and high salinity [11]; similarly *AtNADK3* transcription can be induced by MV, high salinity and osmotic shock [10], [36]. In this study, we found that *AtNADK1* and *OsNADK1/2* belongs to subfamily I and can be induced to varying degrees by cold, drought (PEG), salt (NaCl), oxidative (MV) and pathogens (Figures 6, 7, S6 and S7). We also found that *AtNADK2*, which belongs to subfamily II, is up-regulated by pathogens such as *P. syringae* pv. *phaseolicola* and *Hyaloperonospora arabidopsidis* (Figure S6); *OsNADK3*, which also belongs to subfamily II, is up-regulated by *Nilaparvata lugens* and down-regulated under anaerobic conditions (Figure S6). *OsNADK4* belongs to subfamily III, and is up-regulated under anaerobic conditions, *N. lugens* and heat in root, and slightly up-regulated by drought or PEG in root (Figures 7 and S6). These observations, together with our analysis of *cis*-elements (Table 1), suggest that NADK genes in subfamilies I, II and III play an important role in plant responses to invading pathogens and abiotic stresses. The functions of NADKs in subfamily IV in abiotic/biotic-stress responses remains to be examined.

### NADKs may participate in regulation of hormone signaling

*AtNADK3* transcription can be slightly induced by ABA and the mutant is hypersensitive to ABA [10]. In this study, we found that all *AtNADK1*–*3* showed down-regulated or slightly down-regulated in response to ABA (Figure 6), while *OsNADK1*–*3* were up-regulated by ABA treatment (Figure 7). It’s important to note that response profiles of NADK genes by qRT-PCR are not completely consisted with microarray data or previous studies, because of the different experimental material, processing and analyzing methods used in this study. Moreover, seven AtNADK and OsNADK genes (*AtNADK1–3* and *OsNADK1–4*) contain *cis*-acting ABA responsive elements (Table 1), suggesting that NADKs probably participate in regulation of ABA signaling in plants.

JA and its derivative MeJA are important signaling molecules in plant responses to many abiotic and biotic stresses such as wounding and pathogens [56], [57]. Moreover, JA biosynthesis occurs in peroxisomes [57], [58]. AtNADK3 is also a peroxisomal enzyme and may phosphorylate the NADH derived from β-oxidation to yield NADPH needed for anti-oxidant defense [36]. *AtNADK1/2* and *OsNADK1–4* were up-regulated or slightly up-regulated under MeJA treatment (Figures 6 and 7). Moreover, several of these genes (*AtNADK1/2/3* and *OsNADK1/4*) contain CGTCA-MeJA-responsiveness motifs in their promoter regions (Table 1). These observations suggest that NADKs may also participate in regulation of JA or MeJA signaling in plants.

It should be pointed out that although transcription factors and *cis*-regulatory elements play important roles in regulating gene expression, the relationship is not always direct and the inducibility of expression is often affected by many factors and on multiple levels [59], [60]. For example, ABA-responsive *cis*-regulatory elements were identified in the promoter regions of all seven NADK genes in *Arabidopsis* and rice, but only *OsNADK1*–*3* were up-regulated or slightly up-regulated by ABA treatment (Figures 7 and S7). Moreover, considering the limitations of the PlantCARE program in predicting *cis*-regulatory elements, further experiments are necessary to verify the relationship between the inducible expression profiles of NADK genes and the *cis*-elements within their promoters.

## Materials and Methods

### Data retrieval and identification of NADK genes

The protein sequences of 24 completely or partially sequenced plant genomes representing the eight major plant lineages were retrieved from public databases. All of the protein sequences were the most current non-redundant sequences from the following sources (Table S1): the glaucophyte *Cyanophora paradoxa* from the *Cyanophora* Genome Project (http://cyanophora.rutgers.edu/cyanophora/home.php) [61]; the rhodophytes *Cyanidioschyzon merolae* and *Galdieria sulphuraria* from the *Cyanidioschyzon* Genome Project (http://merolae.biol.s.u-tokyo.ac.jp/) [62] and the *Galdieria* Genome Project (http://genomics.msu.edu/galdieria/) [63], respectively; the chlorophytes *O. tauri* (version 2.0) [64], *O. lucimarinus* (version 2.0) [65], *M. pusilla* strain RCC299 (version 3.0) and strain CCMP1545 (version 3.0) [66], *Chlorella variabilis* NC64A (version 1.0) [67], *Coccomyxa subellipsoidea* C-169 (version 2.0) [68], *C. reinhardtii* (version 4.0) [69] and *V. carteri* (version 2.0) and the bryophyte *P. patens* (version 3.0) [70] and lycophyte *S. moellendorffii* (version 1.1) [71] from the Joint Genome Institute (JGI, http://genome.jgi-psf.org/); for the gymnosperm *P. sitchensis* [72] from NCBI (http://www.ncbi.nlm.nih.gov/, partial sequences only because of its genome is only partially sequenced); the monocots *B. distachyon* (version 1.2) [73], *Setaria italica* (version 2.1) [74] and *S. bicolor* (version 2.1) [75] from JGI, rice (version 7.0) and *Z. mays* (version 5.6) from the Institute for Genomic Research Rice Genome Annotation Project (http://rice.plantbiology.msu.edu/index.shtml) [76] and MaizeSequence (http://www.maizesequence.org/index.html) [77], respectively; the eudicots *P. trichocarpa* (version 2.2) [78] and *Cucumis sativus* (version 1.0) [79] from JGI, *Arabidopsis* (version 10.0) from the Arabidopsis Information Resource (http://www.arabidopsis.org/) [80], *B. rapa* (version 1.5) from the *Brassica* Database (http://brassicadb.org/brad/) [81] and *V. vinifera* (version 1.0) from GenoScope (http://www.genoscope.cns.fr/externe/GenomeBrowser/Vitis/) [82]. All of the above protein sequences were integrated into a local protein database for the subsequent identification of NADK homologs.

To identify the NADK genes and their homologs in the Plantae supergroup, HMMER v3.0 [83], [84] was used to perform an HMM search against the local protein database, using the family-specific NAD_kinase domain (PF01513) HMM profile obtained from the Pfam database [85], [86]. The HMM search was performed with the default parameters and an E-value cutoff of 1e^−5^. If a candidate gene had multiple alternative splice variants, the longest variant was used to represent the candidate protein. The Pfam and SMART [87], [88] databases were employed to detect conserved domains in the candidate proteins, and the search results were refined manually to eliminate partial NADK domains and other potential false positives.

### Sequence alignment and phylogenetic analysis

The NAD_kinase domain sequences of the candidate proteins were aligned using the MUSCLE v3.8 program with the default parameters [89], [90] and the alignments were manually edited using the BioEdit v7.0 program [91]. The rooted maximum-likelihood tree was inferred from the resulting alignments using the Phylip v3.68 package [92] under the γ-corrected Jones–Taylor–Thornton model [93] with default parameters, and the reliability of interior branches was assessed with 1000 bootstrap resamplings. In addition, the MEGA v5.0 program [94] was also used to reconstruct the phylogenetic trees by the neighbor joining, minimal evolution and maximum parsimony methods, and to display the phylogenetic trees.

The rates of non-synonymous substitution (Ka) and synonymous substitution (Ks) were estimated for the orthologous and paralogous gene pairs of NADK family genes using the Codeml program in PAML v4.3 [95] interface tool of PAL2NAL [96], based on the aligned amino acid sequences and the corresponding nucleotide sequences. The duplication and divergence times of each gene pairs were estimated from the Ks of λ substitutions per synonymous site per year as T = Ks/2λ (λ = 6.5×10^−9^) [97], [98].

### Exon/intron structure and conserved motif analysis

The exon/intron structures of individual NADK genes were obtained through the Gene Structure Display Server (http://gsds.cbi.pku.edu.cn) [99] by aligning the coding or cDNA sequences with their corresponding genomic DNA sequences from Phytozome v9.1 (http://www.phytozome.net/) [100]. To illustrate the evolution of introns, gene models were inspected for annotation of introns, and exon/intron boundaries were manually checked. For a subset of genes, predictions pertaining to the types of introns were independently checked using Common Introns Within Orthologous Genes software (http://ciwog.gdcb.iastate.edu/) [101].

The conserved functional motifs within NAD_kinase domains were identified using the MEME v4.9 program (http://meme.sdsc.edu) [102] with the default parameters, and the sequence logos (graphical representations) of these motifs or domains were generated with the WebLogo v3.3 server (http://weblogo.threeplusone.com/create.cgi) [103] based on the results of protein sequence alignments.

### *Cis*-regulatory elements and expression profile analysis

The 1500 bp upstream of the transcription start site of all NADK genes in *Arabidopsis* and rice were obtained from Phytozome v9.1 (http://www.phytozome.net/), and the *cis*-regulatory elements were identified using the PlantCARE program (http://bioinformatics.psb.ugent.be/webtools/plantcare/html/) [104], [105].

ATH1 22 k and Os 51 k microarray data in the Genevestigator V3 database were used to analyze the tissue-specific and inducible expression profiles of NADK genes in *Arabidopsis* and rice [106], respectively. In addition, EST profiles of each NADK gene in *Arabidopsis* and rice were also analyzed by BLASTN search against the corresponding NCBI (http://www.ncbi.nlm.nih.gov/) EST database, with the coding sequences of the individual NADK gene as query. The BLASTN searches were performed with the following criteria: E-value<1e^−10^ and nucleotide identities >95% over 150 bp.

### Plant materials, treatments and quantitative real-time PCR (qRT-PCR) analysis

The *Arabidopsis* (Col-0) seeds were pretreated at 4°C for 3 days and directly sown in Sunshine MVP potting soil, and then grown in growth chambers under 16 h light/8 h dark at 22±1°C; while rice (*Oryza sativa* ssp. *japonica* cv. Dongjin) seeds were pretreated germination at 26±1°C for 4 days and transplanted to 1/2 Hogland nutrient solution, and then grown in growth chambers under 16 h light/8 h dark at 26±1°C. Following two (rice) or four (*Arabidopsis*) weeks of growth, the seedlings were grown under 4°C or 30°C (*Arabidopsis*)/40°C (rice) for 12 h for cold and heat treatments, respectively; submerged in 200 mM NaCl or 20% PEG6000 solutions for 12 h for drought and salt treatments, respectively. For oxidative stress and hormone treatments, solutions of 30 µM MV, 100 µM MeJA and ABA were separately sprayed on seedlings for 12 h. All the MV, MeJA and ABA used for treatments were purchased from Sigma-Aldrich. Samples were collected following treatment and immediately frozen at −80°C.

Total RNA was extracted by using Trizol reagent (Takara, Japan) according to the manufacturer’s instructions and treated with RNase-free DNase I (Invitrogen, USA) for 15 min to remove any DNA contamination. RNA concentration and quality were verified by using the NanoDrop 1000 Spectrophotometers (Thermo, USA), and cDNAs were synthesized by using oligo d(T)_18_ reverse primer from 5 µg of total RNA in a total volume of 20 µL by using EasyScript First-Strand cDNA Synthesis SuperMix (TransGen Biotech, China). qRT-PCR reactions were carried out in 96-well (20 µL) format by using the FastStart Essential DNA Green Master (Roche, Switzerland), and were performed in an CFX96 Touch Real-Time PCR Detection System (BIO-RAD, USA). The reactions were repeated three times and the quantitative analysis used the 2^−ΔΔCT^ method. All gene-specific primers were designed to avoid the conserved region and span introns or cross an exon-exon junction. The detailed primer sequences are shown in Table S5. The *AtTub6* (AT5G12250) and *OsActin1* (accession ID KC140126) were chosen as the internal control in *Arabidopsis* and rice, respectively.

## Supporting Information

Figure S1 **Amino acid sequence alignment of NAD_kinase domains in NADKs.**
(PDF)

Figure S2 **Phylogenetic relationship and domain organization of NADK genes in plants, yeast and humans.**
(PDF)

Figure S3 **Chromosomal locations of NADK family genes in plants.**
(PDF)

Figure S4 **Segmental duplications and duplication and divergence times of NADK family genes in land plants.**
(PDF)

Figure S5 **Developmental expression patterns of NADK family genes in** ***Arabidopsis*** **and rice.**
(PDF)

Figure S6 **Expression patterns of NADK family genes in** ***Arabidopsis*** **and rice under abiotic and biotic stresses.**
(PDF)

Figure S7 **Expression patterns of NADK family genes in** ***Arabidopsis*** **and rice with various hormone treatments.**
(PDF)

Table S1 **The NADK family genes in plants.**
(PDF)

Table S2 ***Cis*** **-element analysis of the** ***AtNADK*** **and** ***OsNADK*** **promoters.**
(PDF)

Table S3 **EST profiles of NADK genes in** ***Arabidopsis*** **.**
(PDF)

Table S4 **EST profiles of NADK genes in rice.**
(PDF)

Table S5 **The gene-specific primers used for qRT-PCR in this study.**
(PDF)
