# Supplementary material for: Genome-Wide Analysis of the NADK Gene Family in Plants
Source: PLoS One. 2014 Jun 26;9(6):e101051. doi: 10.1371/journal.pone.0101051 (PMC4072752; doi:10.1371/journal.pone.0101051)
Supplement: Figure S1 — Amino acid sequence alignment of NAD_kinase domains in NADKs. (PDF) [file pone.0101051.s001.pdf]

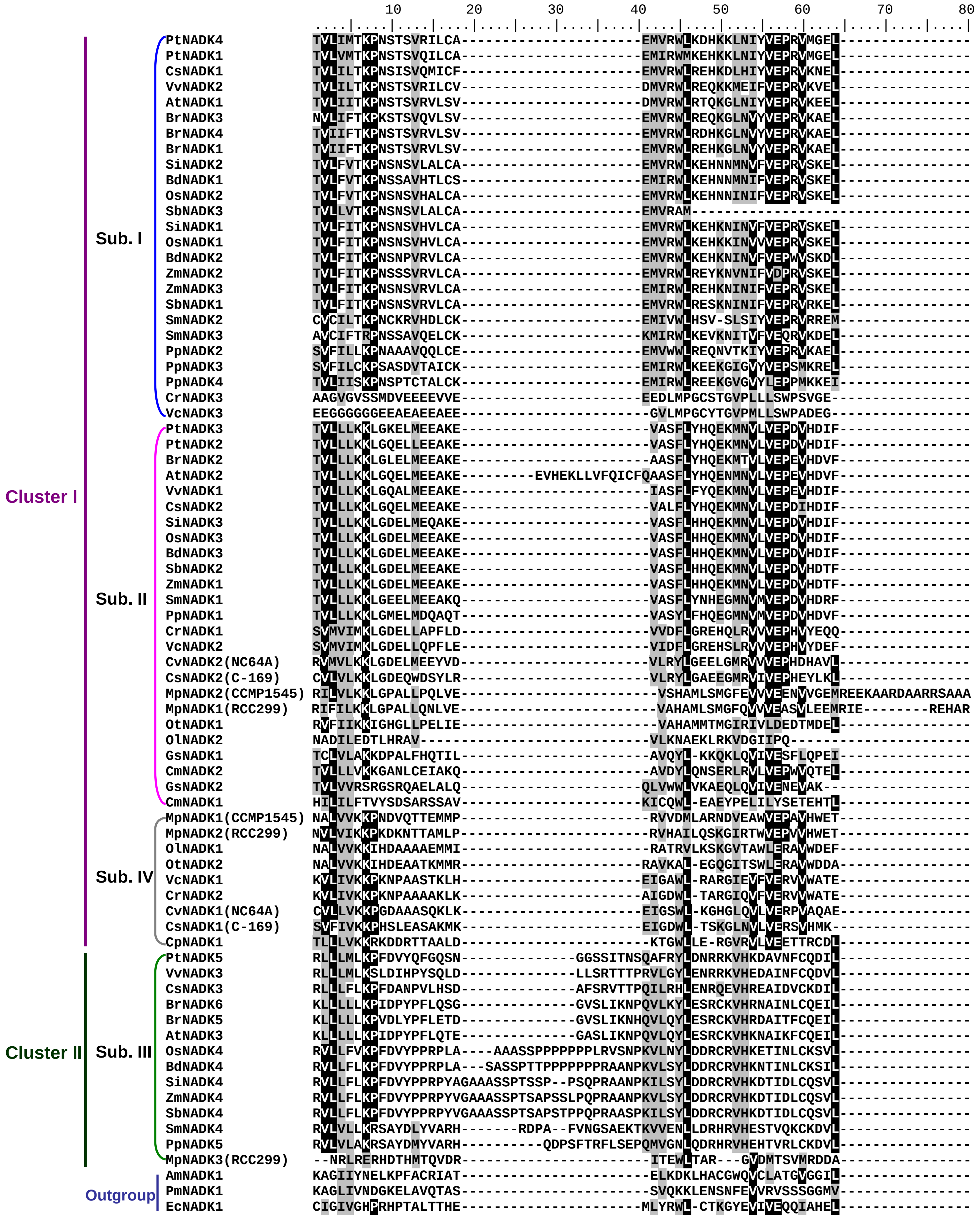

10 20 30 40 50 60 70 80
Cluster I
Cluster II
Outgroup
Sub. I
Sub. II
Sub. IV
Sub. III
PtNADK4 TVLIMTKPNSTSVRILCA--------------------EMVRWLKDHKKLNIYVEPRVMGEL---------------
PtNADK1 TVLVMTKPNSTSVQILCA--------------------EMIRWMKEHKKLNIYVEPRVMGEL---------------
CsNADK1 TVLILTKPNSISVQMICF--------------------EMVRWLREHKKDLHIYVEPRVKNEL---------------
VvNADK2 TVLILTKPNSTSVRILCV--------------------DMVRWLREQKKMEIFVEPRVKVEL---------------
AtNADK1 TVLIITKPNSTSVRVLSV--------------------DMVRWLRTQKGLNIYVEPRVKEEL---------------
BrNADK3 NVLIFTKPKSTSVQVLSV--------------------EMVRWLREQKGLNVYVEPRVKAEL---------------
BrNADK4 TVIIFTKPNSTSVRVLSV--------------------EMVRWLRDHKGLNVYVEPRVKAEL---------------
BrNADK1 TVIIFTKPNSTSVRVLSV--------------------EMVRWLREHKGLNVYVEPRVKAEL---------------
SiNADK2 TVLFVTKPNSNSVLALCA--------------------EMVRWLKEHNNMNVFVEPRVSKEL---------------
BdNADK1 TVLFVTKPNSSAVHTLCS--------------------EMIRWLKEHNNMNIFVEPRVSKEL---------------
OsNADK2 TVLFVTKPNSNSVHALCA--------------------EMVRWLKEHNNINIFVEPRVSKEL---------------
SbNADK3 TVLLVTKPNSNSVLALCA--------------------EMVRAM-------------------------------
SiNADK1 TVLFITKPNSNSVHVLCA--------------------EMVRWLKEHKNINVFVEPRVSKEL---------------
OsNADK1 TVLFITKPNSNSVHVLCA--------------------EMVRWLKEHKKINVVVEPRVSKEL---------------
BdNADK2 TVLFITKPNSNPVRVLCA--------------------EMVRWLKEHKNINVFVEPWVSKDL---------------
ZmNADK2 TVLFITKPNSSSVRVLCA--------------------EMVRWLREYKNVNIFVDPRVSKEL---------------
ZmNADK3 TVLFITKPNSNSVRVLCA--------------------EMIRWLREHKNINIFVEPRVSKEL---------------
SbNADK1 TVLFITKPNSNSVRVLCA--------------------EMVRWLRESKNINIFVEPRVRKEL---------------
SmNADK2 CVCILTKPNCKRVHDLCK--------------------EMIVWLHSV-SLSIYVEPRVRREM---------------
SmNADK3 AVCIFTRPNSSAVQELCK--------------------KMIRWLKEVKNITVFVEQRVKDEL---------------
PpNADK2 SVFILLKPNAAAVQQLCE--------------------EMVWWLREQNVTKIYVEPRVKAEL---------------
PpNADK3 SVFILCKPSASDVTAICK--------------------EMIRWLKEEKGIGVYVEPSMKREL---------------
PpNADK4 TVLIISKPNSPTCTALCK--------------------EMIRWLREEKGVGVYLEPPMKKEI---------------
CrNADK3 AAGVGVSSMDVEEEEVVE--------------------EEDLMPGCSTGVPLLLSWPSVGE----------------
VcNADK3 EEGGGGGGEEAEAEEAEE---------------------GVLMPGCYTGVPMLLSWPADEG----------------
PtNADK3 TVLLLKKLGKELMEEAKE---------------------VASFLYHQEKMNVLVEPDVHDIF---------------
PtNADK2 TVLLLKKLGQELLEEAKE---------------------VASFLYHQEKMNVLVEPDVHDIF---------------
BrNADK2 TVLLLKKLGLELMEEAKE---------------------AASFLYHQEKMTVLVEPEVHDVF---------------
AtNADK2 TVLLLKKLGQELMEEAKE---------EVHEKLLVFQICFQAASFLYHQENMNVLVEPEVHDVF---------------
VvNADK1 TVLLLKKLGQALMEEAKE----------------------IASFLFYQEKMNVLVEPEVHDIF---------------
CsNADK2 TVLLLKKLGQELMEEAKE---------------------VALFLYHQEKMNVLVEPDIHDIF---------------
SiNADK3 TVLLLKKLGDELMEQAKE---------------------VASFLHHQEKMNVLVEPDVHDIF---------------
OsNADK3 TVLLLKKLGDELMEEAKE---------------------VASFLHHQEKMNVLVEPDVHDIF---------------
BdNADK3 TVLLLKKLGDELMEEAKE---------------------VASFLHHQEKMNVLVEPDVHDIF---------------
SbNADK2 TVLLLKKLGDELMEEAKE---------------------VASFLHHQEKMNVLVEPDVHDTF---------------
ZmNADK1 TVLLLKKLGDELMEEAKE---------------------VASFLHHQEKMNVLVEPDVHDTF---------------
SmNADK1 TVLLLKKLGEELMEEAKQ---------------------VASFLYNHEGMNVMVEPDVHDRF---------------
PpNADK1 TVLLLKKLGMELMDQAQT---------------------VASYLFHQEGMNVMVEPDVHDVF---------------
CrNADK1 SVMVIMKLGDELLAPFLD---------------------VVDFLGREHQLRVVVEPHVYEQQ---------------
VcNADK2 SVMVIMKLGDELLQPFLE---------------------VIDFLGREHSLRVVVEPHVYDEF---------------
CvNADK2(NC64A) RVMVLKKLGDELMEEYVD---------------------VLRYLGEELGMRVVVEPHDHAVL---------------
CsNADK2(C-169) CVLVLKKLGDEQWDSYLR---------------------VLRYLGAEEGMRVIVEPHEYLKL---------------
MpNADK2(CCMP1545) RILVLKKLGPALLPQLVE----------------------VSHAMLSMGFEVVVENVVGEMREEKAARDAARRSAAA
MpNADK1(RCC299) RIFILKKLGPALLQNLVE----------------------VAHAMLSMGFQVVVEASVLEEMRIE-------REHAR
OtNADK1 RVFIIKKIGHGLLPELIE----------------------VAHAMMTMGIRIVLDEDTMDEL---------------
OlNADK2 NADILEDTLHRAV-------------------------VLKNAEKLRKVDGIIPQ-------------------
GsNADK1 TCLVLAKKDPALFHQTIL---------------------AVQYL-KKQKLQVIVESFLQPEI---------------
CmNADK2 TVLLLVKKGANLCEIAKQ---------------------AVDYLQNSERLRVLVEPWVQTEL---------------
GsNADK2 TVLVVRSRGSRQAELALQ--------------------QLVWWLVKAEQLQVIVENEVAK-----------------
CmNADK1 HILILFTVYSDSARSSAV--------------------KICQWL-EAEYPELILYSETEHTL---------------
MpNADK1(CCMP1545) NALVVKKPNDVQTTEMMP---------------------RVVDMLARNDVEAWVEPAVHWET---------------
MpNADK2(RCC299) NVLVIKKPKDKNTTAMLP---------------------RVHAILQSKGIRTWVEPVVHWET---------------
OlNADK1 NALVVKKIHDAAAAEMMI---------------------RATRVLKSKGVTAWLERAVWDEF---------------
OtNADK2 NALVVKKIHDEAATKMMR--------------------RAVKAL-EGQGITSWLERAVWDDA---------------
VcNADK1 KVLIVKKPKNPAASTKLH--------------------EIGAWL-RARGIEVFVERVVWATE---------------
CrNADK2 KVLIVKKPKNPAAAAKLK--------------------AIGDWL-TARGIQVFVERVVWATE---------------
CvNADK1(NC64A) CVLLVKKPGDAAASQKLK--------------------EIGSWL-KGHGLQVLVERPVAQAE---------------
CsNADK1(C-169) SVFIVKKPHSLEASAKMK--------------------EIGDWL-TSKGLNVLVERSVHMK----------------
CpNADK1 TLLLVKKRKDDRTTAALD---------------------KTGWLLE-RGVRVLVEETTRCDL---------------
PtNADK5 RLLLMLKPFDVYQFGQSN-------------GGSSITNSQAFRYLDNRRKVHKDAVNFCQDIL---------------
VvNADK3 RLLLMLKSLDIHPYSQLD-------------LLSRTTTPRVLGYLENRRKVHEDAINFCQDVL---------------
CsNADK3 RLLLFLKPFDANPVLHSD-------------AFSRVTTPQILRHLENRQEVHREAIDVCKDIL---------------
BrNADK6 KLLLLLKPIDPYPFLQSG-------------GVSLIKNPQVLKYLESRCKVHRNAINLCQEIL---------------
BrNADK5 KLLLLLKPVDLYPFLETD-------------GVSLIKNHQVLQYLESRCKVHRDAITFCQEIL---------------
AtNADK3 KLLLLLKPIDPYPFLQTE-------------GASLIKNPQVLQYLESRCKVHKNAIKFCQEIL---------------
OsNADK4 RVLLFVKPFDVYPPRPLA---AAASSPPPPPPPLRVSNPKVLNYLDDRCRVHKETINLCKSVL---------------
BdNADK4 RVLLFLKPFDVYPPRPLA---SASSPTTPPPPPPPRAANPKVLSYLDDRCRVHKNTINLCKSIL---------------
SiNADK4 RVLLFLKPFDVYPPRPYAGAAASSPTSSP--PSQPRAANPKILSYLDDRCRVHKDTIDLCQSVL---------------
ZmNADK4 RVLLFLKPFDVYPPRPYVGAAASSPTSAPSSLPQPRAANPKVLSYLDDRCRVHKDTIDLCQSVL---------------
SbNADK4 RVLLFLKPFDVYPPRPYVGAAASSPTSAPSTPPQPRAASPKILSYLDDRCRVHKDTIDLCQSVL---------------
SmNADK4 RVLVLLKRSAYDLYVARH-------RDPA--FVNGSAEKTKVVENLLDRHRVHESTVQKCKDVL---------------
PpNADK5 RVLVLAKRSAYDMYVARH----------QDPSFTRFLSEPQMVGNLQDRHRVHEHTVRLCKDVL---------------
MpNADK3(RCC299) --NRLRERHDTHMTQVDR---------------------ITEWLTAR---GVDMTSVMRDDA---------------
AmNADK1 KAGIIYNELKPFACRIAT---------------------ELKDKLHACGWQVCLATGVGGIL---------------
PmNADK1 KAGLIVNDGKELAVQTAS---------------------SVQKKLENSNFEVVRVSSSGGMV---------------
EcNADK1 CIGIVGHPRHPTALTTHE--------------------MLYRWL-CTKGYEVIVEQQIAHEL---------------

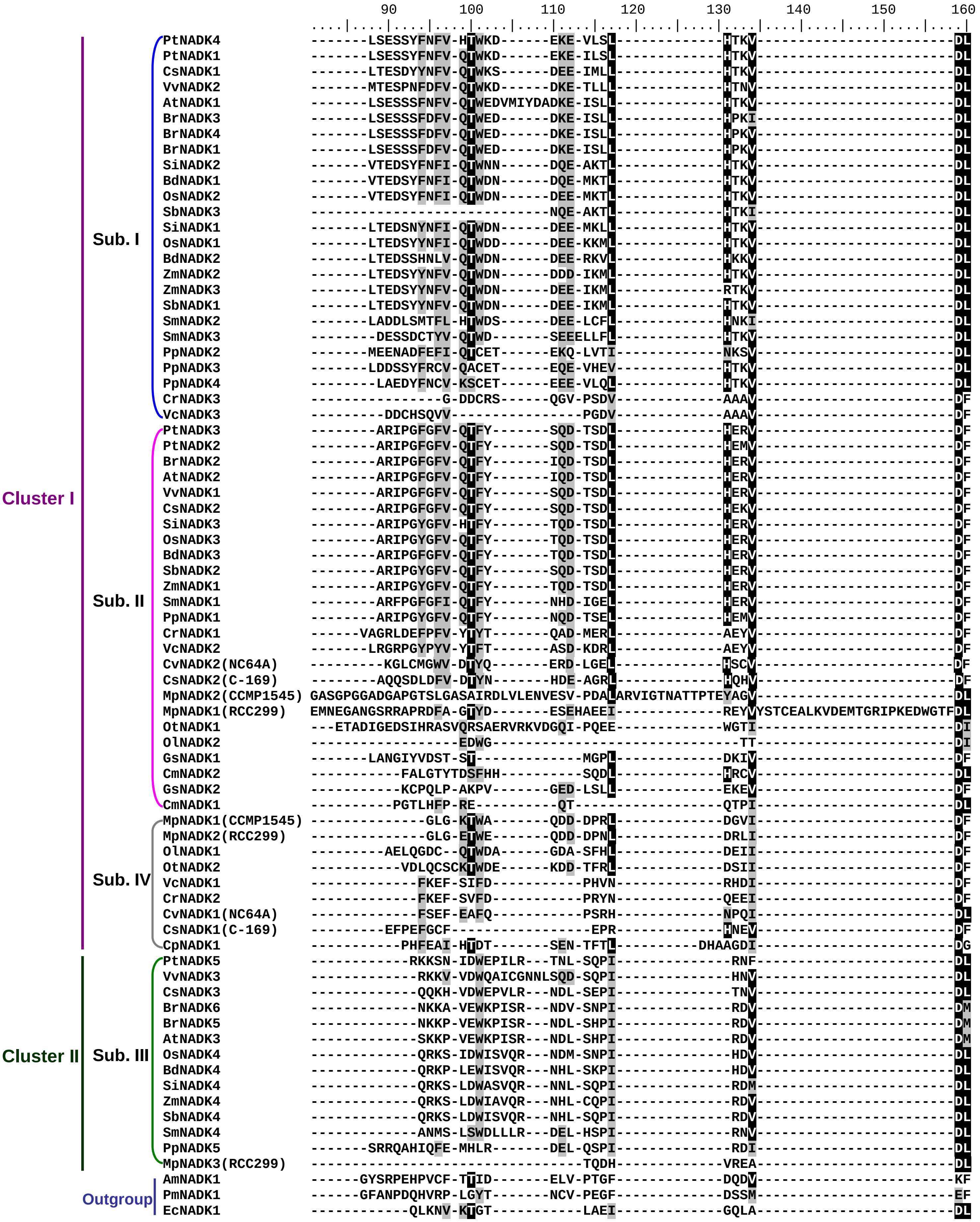
90 100 110 120 130 140 150 160
Cluster I
Sub. I
PtNADK4 -------LSESSYFNFV-HTWKD------EKE-VLSL-------------HTKV------------------------DL
PtNADK1 -------LSESSYFNFV-QTWKD------EKE-ILSL-------------HTKV------------------------DL
CsNADK1 -------LTESDYYNFV-QTWKS------DEE-IMLL-------------HTKV------------------------DL
VvNADK2 -------MTESPNFDFV-QTWKD------DKE-TLLL-------------HTNV------------------------DL
AtNADK1 -------LSESSSFNFV-QTWEDVMIYDADKE-ISLL-------------HTKV------------------------DL
BrNADK3 -------LSESSSFDFV-QTWED------DKE-ISLL-------------HPKI------------------------DL
BrNADK4 -------LSESSSFDFV-QTWED------DKE-ISLL-------------HPKV------------------------DL
BrNADK1 -------LSESSSFDFV-QTWED------DKE-ISLL-------------HPKV------------------------DL
SiNADK2 -------VTEDSYFNFI-QTWNN------DQE-AKTL-------------HTKV------------------------DL
BdNADK1 -------VTEDSYFNFI-QTWDN------DQE-MKTL-------------HTKV------------------------DL
OsNADK2 -------VTEDSYFNFI-QTWDN------DEE-MKTL-------------HTKV------------------------DL
SbNADK3 -----------------------------NQE-AKTL-------------HTKI------------------------DL
SiNADK1 -------LTEDSNYNFI-QTWDN------DEE-MKLL-------------HTKV------------------------DL
OsNADK1 -------LTEDSYYNFI-QTWDD------DEE-KKML-------------HTKV------------------------DL
BdNADK2 -------LTEDSSHNLV-QTWDN------DEE-RKVL-------------HKKV------------------------DL
ZmNADK2 -------LTEDSYYNFV-QTWDN------DDD-IKML-------------HTKV------------------------DL
ZmNADK3 -------LTEDSYYNFV-QTWDN------DEE-IKML-------------RTKV------------------------DL
SbNADK1 -------LTEDSYYNFV-QTWDN------DEE-IKML-------------HTKV------------------------DL
SmNADK2 -------LADDLSMTFL-HTWDS------DEE-LCFL-------------HNKI------------------------DL
SmNADK3 --------DESSDCTYV-QTWD-------SEEELLFL-------------HTKV------------------------DL
PpNADK2 -------MEENADFEFI-QTCET------EKQ-LVTI-------------NKSV------------------------DL
PpNADK3 -------LDDSSYFRCV-QACET------EQE-VHEV-------------HTKV------------------------DL
PpNADK4 --------LAEDYFNCV-KSCET------EEE-VLQL-------------HTKV------------------------DL
CrNADK3 ---------------G-DDCRS------QGV-PSDV-------------AAAV------------------------DF
VcNADK3 ---------DDCHSQVV-----------------PGDV-------------AAAV------------------------DF
Sub. II
PtNADK3 --------ARIPGFGFV-QTFY-------SQD-TSDL-------------HERV------------------------DF
PtNADK2 --------ARIPGFGFV-QTFY-------SQD-TSDL-------------HEMV------------------------DF
BrNADK2 --------ARIPGFGFV-QTFY-------IQD-TSDL-------------HERV------------------------DF
AtNADK2 --------ARIPGFGFV-QTFY-------IQD-TSDL-------------HERV------------------------DF
VvNADK1 --------ARIPGFGFV-QTFY-------SQD-TSDL-------------HERV------------------------DF
CsNADK2 --------ARIPGFGFV-QTFY-------SQD-TSDL-------------HEKV------------------------DF
SiNADK3 --------ARIPGYGFV-HTFY-------TQD-TSDL-------------HERV------------------------DF
OsNADK3 --------ARIPGYGFV-QTFY-------TQD-TSDL-------------HERV------------------------DF
BdNADK3 --------ARIPGFGFV-QTFY-------TQD-TSDL-------------HERV------------------------DF
SbNADK2 --------ARIPGYGFV-QTFY-------SQD-TSDL-------------HERV------------------------DF
ZmNADK1 --------ARIPGYGFV-QTFY-------TQD-TSDL-------------HERV------------------------DF
SmNADK1 --------ARFPGFGFI-QTFY-------NHD-IGEL-------------HERV------------------------DF
PpNADK1 --------ARIPGYGFV-QTFY-------NQD-TSEL-------------HEMV------------------------DF
CrNADK1 ------VAGRLDEFPFV-YTYT-------QAD-MERL-------------AEYV------------------------DF
VcNADK2 -------LRGRPGYPYV-YTFT-------ASD-KDRL-------------AEYV------------------------DF
CvNADK2(NC64A) ---------KGLCMGWV-DTYQ-------ERD-LGEL-------------HSCV------------------------DF
CsNADK2(C-169) --------AQQSDLDFV-DTYN-------HDE-AGRL-------------HQHV------------------------DF
MpNADK2(CCMP1545) GASGPGGADGAPGTSLGASAIRDLVLENVESV-PDALARVIGTNATTPTEYAGV------------------------DL
MpNADK1(RCC299) EMNEGANGSRRAPRDFA-GTYD-------ESEHAEEI-------------REYVYSTCEALKVDEMTGRIPKEDWGTFDL
OtNADK1 ---ETADIGEDSIHRASVQRSAERVRKVDGQI-PQEE-------------WGTI------------------------DI
OlNADK2 ------------------EDWG--------------------------------TT------------------------DI
GsNADK1 -------LANGIYVDST-ST-------------MGPL-------------DKIV------------------------DF
CmNADK2 -----------FALGTYTDSFHH----------SQDL-------------HRCV------------------------DL
GsNADK2 -----------KCPQLP-AKPV-------GED-LSLL-------------EKEV------------------------DF
CmNADK1 ----------PGTLHFP-RE----------QT------------------QTPI------------------------DL
Sub. IV
MpNADK1(CCMP1545) --------------GLG-KTWA-------QDD-DPRL-------------DGVI------------------------DF
MpNADK2(RCC299) --------------GLG-ETWE-------QDD-DPNL-------------DRLI------------------------DF
OlNADK1 ---------AELQGDC--QTWDA------GDA-SFHL-------------DEII------------------------DF
OtNADK2 -----------VDLQCSCKTWDE------KDD-TFRL-------------DSII------------------------DF
VcNADK1 -------------FKEF-SIFD-----------PHVN-------------RHDI------------------------DF
CrNADK2 -------------FKEF-SVFD-----------PRYN-------------QEEI------------------------DF
CvNADK1(NC64A) -------------FSEF-EAFQ-----------PSRH-------------NPQI------------------------DL
CsNADK1(C-169) ---------EFPEFGCF-----------------EPR--------------HNEV------------------------DF
CpNADK1 -----------PHFEAI-HTDT-------SEN-TFTL----------DHAAGDI------------------------DG
Cluster II
Sub. III
PtNADK5 ------------RKKSN-IDWEPILR---TNL-SQPI--------------RNF------------------------DL
VvNADK3 ------------RKKV-VDWQAICGNNLSQD-SQPI--------------HNV------------------------DL
CsNADK3 -------------QQKH-VDWEPVLR---NDL-SEPI--------------TNV------------------------DL
BrNADK6 -------------NKKA-VEWKPISR---NDV-SNPI--------------RDV------------------------DM
BrNADK5 -------------NKKP-VEWKPISR---NDL-SHPI--------------RDV------------------------DM
AtNADK3 -------------SKKP-VEWKPISR---NDL-SHPI--------------RDV------------------------DM
OsNADK4 -------------QRKS-IDWISVQR---NDM-SNPI--------------HDV------------------------DL
BdNADK4 -------------QRKP-LEWISVQR---NHL-SKPI--------------HDV------------------------DL
SiNADK4 -------------QRKS-LDWASVQR---NNL-SQPI--------------RDM------------------------DL
ZmNADK4 -------------QRKS-LDWIAVQR---NHL-CQPI--------------RDV------------------------DL
SbNADK4 -------------QRKS-LDWISVQR---NHL-SQPI--------------RDV------------------------DL
SmNADK4 -------------ANMS-LSWDLLLR---DEL-HSPI--------------RNV------------------------DL
PpNADK5 -------SRRQAHIQFE-MHLR-------DEL-QSPI--------------RDI------------------------DL
MpNADK3(RCC299) ---------------------------------TQDH-------------VREA------------------------DL
Outgroup
AmNADK1 ------GYSRPEHPVCF-TTID-------ELV-PTGF-------------DQDV------------------------KF
PmNADK1 ------GFANPDQHVRP-LGYT-------NCV-PEGF-------------DSSM------------------------EF
EcNADK1 ------------QLKNV-KTGT-----------LAEI-------------GQLA------------------------DL

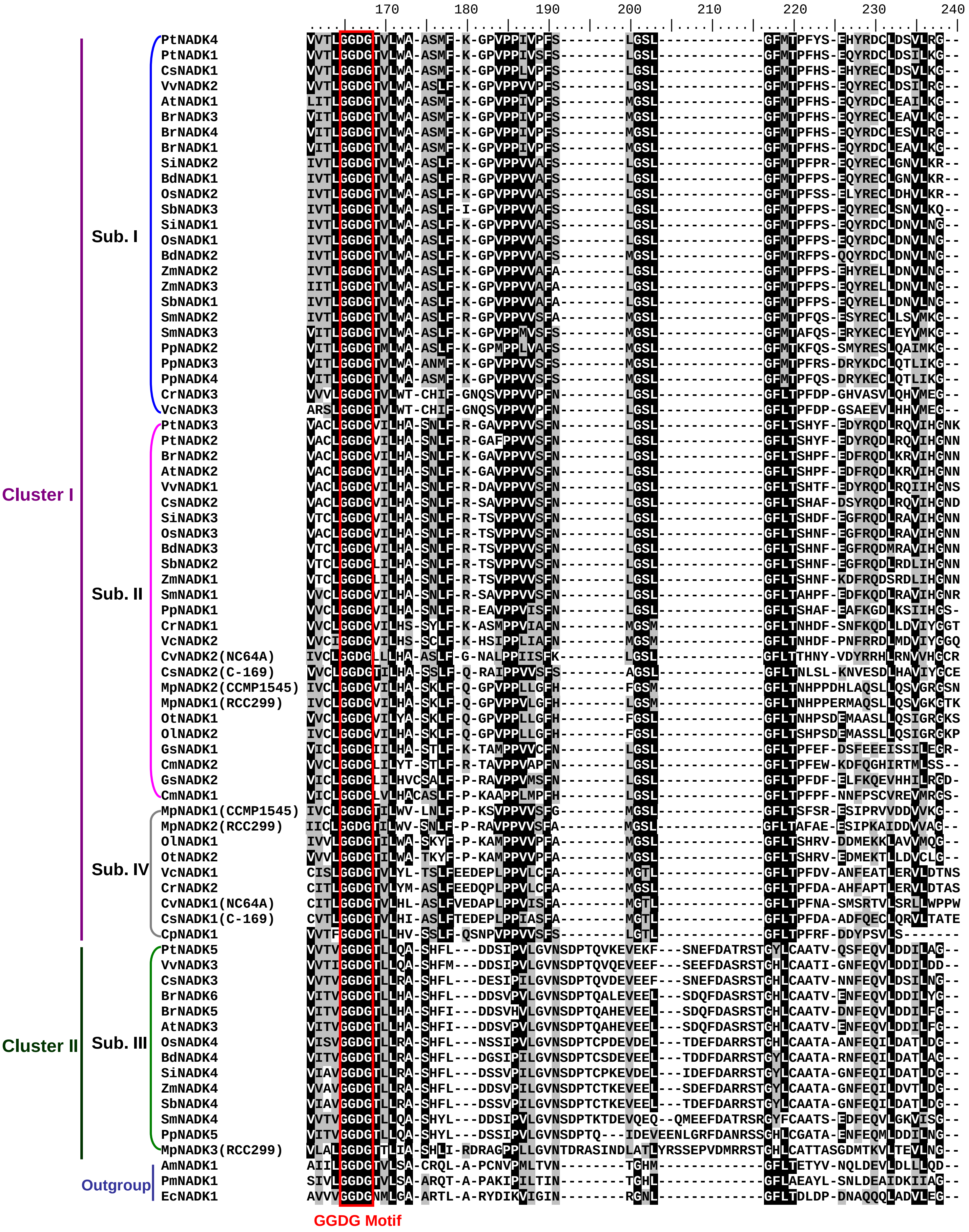
170 180 190 200 210 220 230 240
Cluster I
Sub. I
PtNADK4
PtNADK1
CsNADK1
VvNADK2
AtNADK1
BrNADK3
BrNADK4
BrNADK1
SiNADK2
BdNADK1
OsNADK2
SbNADK3
SiNADK1
OsNADK1
BdNADK2
ZmNADK2
ZmNADK3
SbNADK1
SmNADK2
SmNADK3
PpNADK2
PpNADK3
PpNADK4
CrNADK3
VcNADK3
Sub. II
PtNADK3
PtNADK2
BrNADK2
AtNADK2
VvNADK1
CsNADK2
SiNADK3
OsNADK3
BdNADK3
SbNADK2
ZmNADK1
SmNADK1
PpNADK1
CrNADK1
VcNADK2
CvNADK2(NC64A)
CsNADK2(C-169)
MpNADK2(CCMP1545)
MpNADK1(RCC299)
OtNADK1
OlNADK2
GsNADK1
CmNADK2
GsNADK2
CmNADK1
Sub. IV
MpNADK1(CCMP1545)
MpNADK2(RCC299)
OlNADK1
OtNADK2
VcNADK1
CrNADK2
CvNADK1(NC64A)
CsNADK1(C-169)
CpNADK1
Cluster II
Sub. III
PtNADK5
VvNADK3
CsNADK3
BrNADK6
BrNADK5
AtNADK3
OsNADK4
BdNADK4
SiNADK4
ZmNADK4
SbNADK4
SmNADK4
PpNADK5
MpNADK3(RCC299)
Outgroup
AmNADK1
PmNADK1
EcNADK1
GGDG Motif

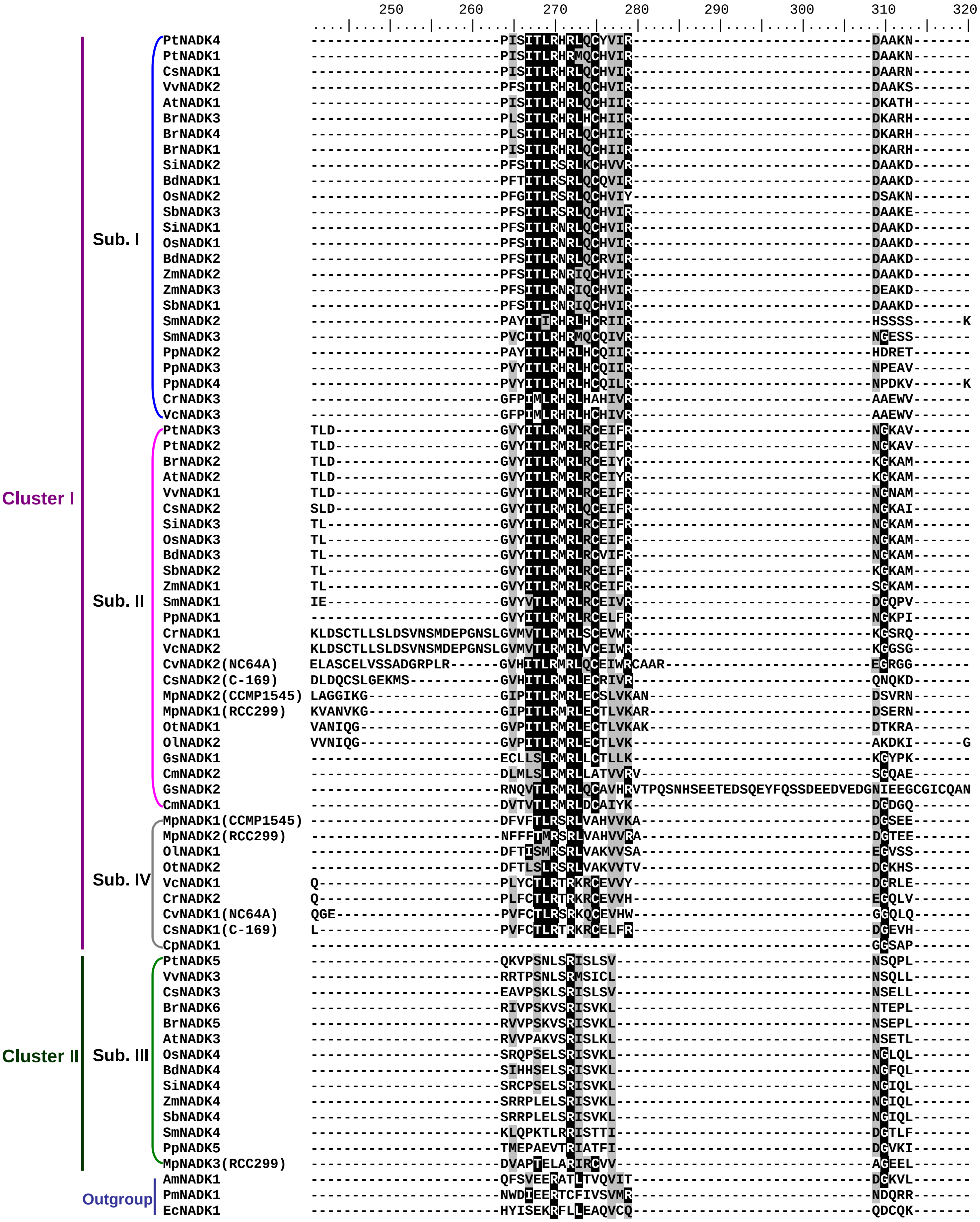
250 260 270 280 290 300 310 320
Cluster I
Sub. I
PtNADK4 -----------------------PISITLRHRLQCYVIR-----------------------------DAAKN-------
PtNADK1 -----------------------PISITLRHRMQCHVIR-----------------------------DAAKN-------
CsNADK1 -----------------------PISITLRHRLQCHVIR-----------------------------DAARN-------
VvNADK2 -----------------------PFSITLRHRLQCHVIR-----------------------------DAAKS-------
AtNADK1 -----------------------PISITLRHRLQCHIIR-----------------------------DKATH-------
BrNADK3 -----------------------PLSITLRHRLHCHIIR-----------------------------DKARH-------
BrNADK4 -----------------------PLSITLRHRLQCHIIR-----------------------------DKARH-------
BrNADK1 -----------------------PISITLRHRLQCHIIR-----------------------------DKARH-------
SiNADK2 -----------------------PFSITLRSRLKCHVVR-----------------------------DAAKD-------
BdNADK1 -----------------------PFTITLRSRLQCQVIR-----------------------------DAAKD-------
OsNADK2 -----------------------PFGITLRSRLQCHVIY-----------------------------DSAKN-------
SbNADK3 -----------------------PFSITLRSRLQCHVIR-----------------------------DAAKE-------
SiNADK1 -----------------------PFSITLRNRLQCHVIR-----------------------------DAAKD-------
OsNADK1 -----------------------PFSITLRNRLQCHVIR-----------------------------DAAKD-------
BdNADK2 -----------------------PFSITLRNRLQCRVIR-----------------------------DAAKD-------
ZmNADK2 -----------------------PFSITLRNRIQCHVIR-----------------------------DAAKD-------
ZmNADK3 -----------------------PFSITLRNRIQCHVIR-----------------------------DEAKD-------
SbNADK1 -----------------------PFSITLRNRIQCHVIR-----------------------------DAAKD-------
SmNADK2 -----------------------PAYITIRHRLHCRIIR-----------------------------HSSSS------K
SmNADK3 -----------------------PVCITLRHRMQCQIVR-----------------------------NGESS-------
PpNADK2 -----------------------PAYITLRHRLHCQIIR-----------------------------HDRET-------
PpNADK3 -----------------------PVYITLRHRLHCQIIR-----------------------------NPEAV-------
PpNADK4 -----------------------PVYITLRHRLHCQILR-----------------------------NPDKV------K
CrNADK3 -----------------------GFPIMLRHRLHAHIVR-----------------------------AAEWV-------
VcNADK3 -----------------------GFPIMLRHRLHCHIVR-----------------------------AAEWV-------
Sub. II
PtNADK3 TLD--------------------GVYITLRMRLRCEIFR-----------------------------NGKAV-------
PtNADK2 TLD--------------------GVYITLRMRLRCEIFR-----------------------------NGKAV-------
BrNADK2 TLD--------------------GVYITLRMRLRCEIYR-----------------------------KGKAM-------
AtNADK2 TLD--------------------GVYITLRMRLRCEIYR-----------------------------KGKAM-------
VvNADK1 TLD--------------------GVYITLRMRLRCEIFR-----------------------------NGNAM-------
CsNADK2 SLD--------------------GVYITLRMRLQCEIFR-----------------------------NGKAI-------
SiNADK3 TL---------------------GVYITLRMRLRCEIFR-----------------------------NGKAM-------
OsNADK3 TL---------------------GVYITLRMRLRCEIFR-----------------------------NGKAM-------
BdNADK3 TL---------------------GVYITLRMRLRCVIFR-----------------------------NGKAM-------
SbNADK2 TL---------------------GVYITLRMRLRCEIFR-----------------------------KGKAM-------
ZmNADK1 TL---------------------GVYITLRMRLRCEIFR-----------------------------SGKAM-------
SmNADK1 IE---------------------GVYVTLRMRLRCEIVR-----------------------------DGQPV-------
PpNADK1 -----------------------GVYITLRMRLRCELFR-----------------------------NGKPI-------
CrNADK1 KLDSCTLLSLDSVNSMDEPGNSLGVMVTLRMRLSCEVWR-----------------------------KGSRQ-------
VcNADK2 KLDSCTLLSLDSVNSMDEPGNSLGVMVTLRMRLVCEIWR-----------------------------KGGSG-------
CvNADK2(NC64A) ELASCELVSSADGRPLR------GVHITLRMRLQCEIWRCAAR-------------------------EGRGG-------
CsNADK2(C-169) DLDQCSLGEKMS-----------GVHITLRMRLECRIVR-----------------------------QNQKD-------
MpNADK2(CCMP1545) LAGGIKG----------------GIPITLRMRLECSLVKAN---------------------------DSVRN-------
MpNADK1(RCC299) KVANVKG----------------GIPITLRMRLECTLVKAR---------------------------DSERN-------
OtNADK1 VANIQG-----------------GVPITLRMRLECTLVKAK---------------------------DTKRA-------
OlNADK2 VVNIQG-----------------GVPITLRMRLECTLVK-----------------------------AKDKI------G
GsNADK1 -----------------------ECLLSLRMRLLCTLLK-----------------------------KGYPK-------
CmNADK2 -----------------------DLMLSLRMRLLATVVRV----------------------------SGQAE-------
GsNADK2 -----------------------RNQVTLRMRLQCAVHRVTPQSNHSEETEDSQEYFQSSDEEDVEDGNIEEGCGICQAN
CmNADK1 -----------------------DVTVTLRMRLDCAIYK-----------------------------DGDGQ-------
Sub. IV
MpNADK1(CCMP1545) -----------------------DFVFTLRSRLVAHVVKA----------------------------DGSEE-------
MpNADK2(RCC299) -----------------------NFFFTMRSRLVAHVVRA----------------------------DGTEE-------
OlNADK1 -----------------------DFTISMRSRLVAKVVSA----------------------------EGVSS-------
OtNADK2 -----------------------DFTLSLRSRLVAKVVTV----------------------------DGKHS-------
VcNADK1 Q----------------------PLYCTLRTRKRCEVVY-----------------------------DGRLE-------
CrNADK2 Q----------------------PLFCTLRTRKRCEVVH-----------------------------EGQLV-------
CvNADK1(NC64A) QGE--------------------PVFCTLRSRKQCEVHW-----------------------------GGQLQ-------
CsNADK1(C-169) L----------------------PVFCTLRTRKRCELFR-----------------------------DGEVH-------
CpNADK1 --GGSAP-------
Cluster II
Sub. III
PtNADK5 -----------------------QKVPSNLSRISLSV-------------------------------NSQPL-------
VvNADK3 -----------------------RRTPSNLSRMSICL-------------------------------NSQLL-------
CsNADK3 -----------------------EAVPSKLSRISLSV-------------------------------NSELL-------
BrNADK6 -----------------------RIVPSKVSRISVKL-------------------------------NTEPL-------
BrNADK5 -----------------------RVVPSKVSRISVKL-------------------------------NSEPL-------
AtNADK3 -----------------------RVVPAKVSRISLKL-------------------------------NSETL-------
OsNADK4 -----------------------SRQPSELSRISVKL-------------------------------NGLQL-------
BdNADK4 -----------------------SIHHSELSRISVKL-------------------------------NGFQL-------
SiNADK4 -----------------------SRCPSELSRISVKL-------------------------------NGIQL-------
ZmNADK4 -----------------------SRRPLELSRISVKL-------------------------------NGIQL-------
SbNADK4 -----------------------SRRPLELSRISVKL-------------------------------NGIQL-------
SmNADK4 -----------------------KLQPKTLRRISTTI-------------------------------DGTLF-------
PpNADK5 -----------------------TMEPAEVTRIATFI-------------------------------DGVKI-------
MpNADK3(RCC299) -----------------------DVAPTELARIRCVV-------------------------------AGEEL-------
Outgroup
AmNADK1 -----------------------QFSVEERATLTVQVIT-----------------------------DGKVL-------
PmNADK1 -----------------------NWDIEERTCFIVSVMR-----------------------------NDQRR-------
EcNADK1 -----------------------HYISEKRFLLEAQVCQ-----------------------------QDCQK-------

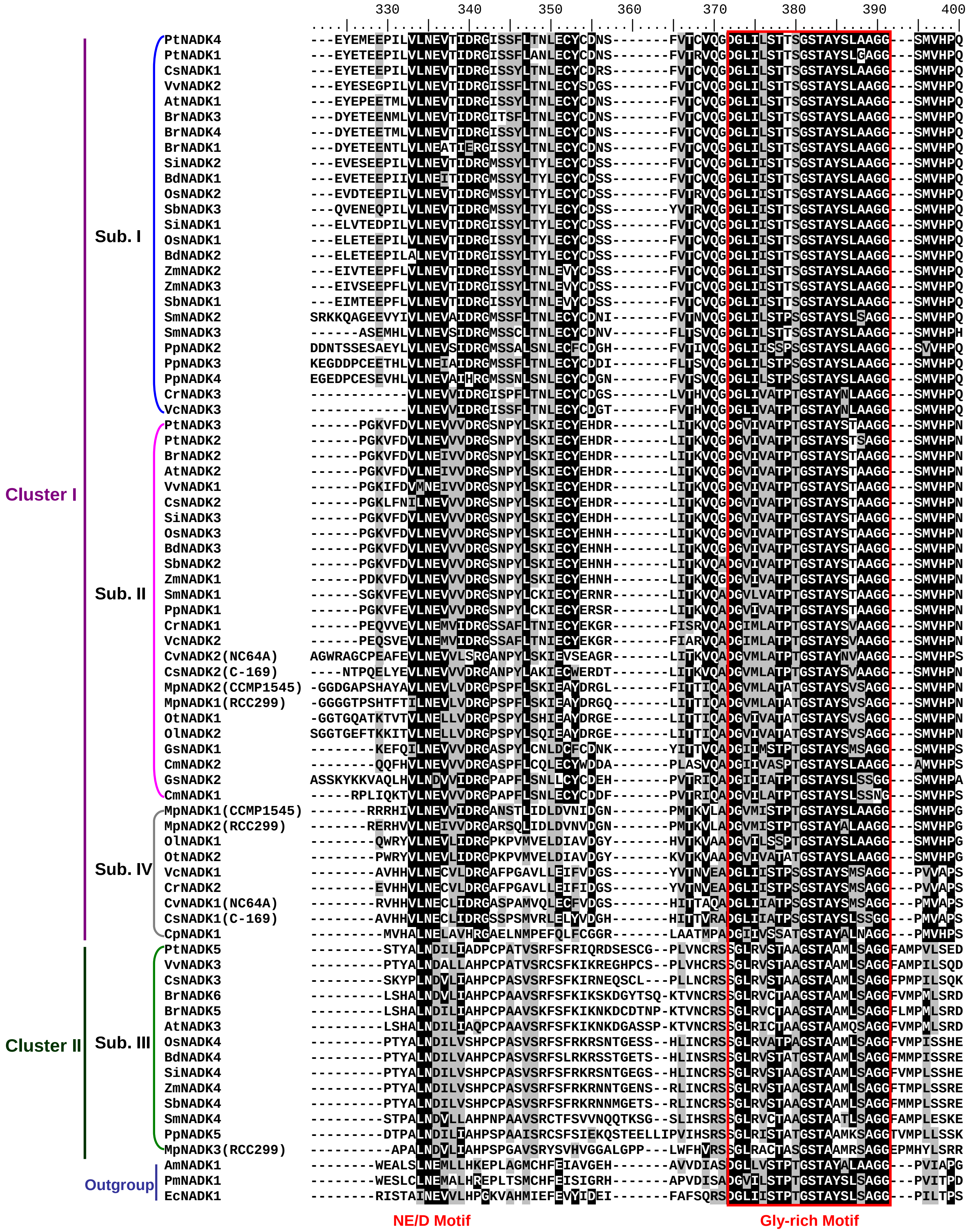
330 340 350 360 370 380 390 400
Cluster I
Sub. I
Sub. II
Sub. IV
Cluster II
Sub. III
Outgroup
PtNADK4 ---EYEMEEPILVLNEVTIDRGISSFLTNLECYCDNS------FVTCVQGDGLILSTTSGSTAYSLAAGG---SMVHPQ
PtNADK1 ---EYETEEPILVLNEVTIDRGISSFLANLECYCDNS------FVTRVQGDGLILSTTSGSTAYSLGAGG---SMVHPQ
CsNADK1 ---EYETEEPILVLNEVTIDRGISSYLTNLECYCDRS------FVTCVQGDGLILSTTSGSTAYSLAAGG---SMVHPQ
VvNADK2 ---EYESEGPILVLNEVTIDRGISSFLTNLECYSDGS------FVTCVQGDGLILSTTSGSTAYSLAAGG---SMVHPQ
AtNADK1 ---EYEPEETMLVLNEVTIDRGISSYLTNLECYCDNS------FVTCVQGDGLILSTTSGSTAYSLAAGG---SMVHPQ
BrNADK3 ---DYETEENMLVLNEVTIDRGITSFLTNLECYCDNS------FVTCVQGDGLILSTTSGSTAYSLAAGG---SMVHPQ
BrNADK4 ---DYETEETMLVLNEVTIDRGISSYLTNLECYCDNS------FVTCVQGDGLILSTTSGSTAYSLAAGG---SMVHPQ
BrNADK1 ---DYETEENTLVLNEATIERGISSYLTNLECYCDNS------FVTCVQGDGLILSTTSGSTAYSLAAGG---SMVHPQ
SiNADK2 ---EVESEEPILVLNEVTIDRGMSSYLTYLECYCDSS------FVTCVQGDGLIISTTSGSTAYSLAAGG---SMVHPQ
BdNADK1 ---EVETEEPIIVLNEITIDRGMSSYLTYLECYCDSS------FVTCVQGDGLIISTTSGSTAYSLAAGG---SMVHPQ
OsNADK2 ---EVDTEEPILVLNEVTIDRGMSSYLTYLECYCDSS------FVTRVQGDGLIISTTSGSTAYSLAAGG---SMVHPQ
SbNADK3 ---QVENEQPILVLNEVTIDRGMSSYLTYLECYCDSS------YVTRVQGDGLIISTTSGSTAYSLAAGG---SMVHPQ
SiNADK1 ---ELVTEDPILVLNEVTIDRGISSYLTYLECYCDSS------FVTCVQGDGLIISTTSGSTAYSLAAGG---SMVHPQ
OsNADK1 ---ELETEEPILVLNEVTIDRGISSYLTYLECYCDSS------FVTCVQGDGLIISTTSGSTAYSLAAGG---SMVHPQ
BdNADK2 ---ELETEEPILALNEVTIDRGISSYLTYLECYCDSS------FVTCVQGDGLIISTTSGSTAYSLAAGG---SMVHPQ
ZmNADK2 ---EIVTEEPFLVLNEVTIDRGISSYLTNLEVYCDSS------FVTCVQGDGLIISTTSGSTAYSLAAGG---SMVHPQ
ZmNADK3 ---EIVSEEPFLVLNEVTIDRGISSYLTNLEVYCDSS------FVTCVQGDGLIISTTSGSTAYSLAAGG---SMVHPQ
SbNADK1 ---EIMTEEPFLVLNEVTIDRGISSYLTNLEVYCDSS------FVTCVQGDGLIISTTSGSTAYSLAAGG---SMVHPQ
SmNADK2 SRKKQAGEEVYIVLNEVAIDRGMSSFLTNLECYCDNI------FVTNVQGDGLILSTPSGSTAYSLSAGG---SMVHPQ
SmNADK3 ------ASEMHLVLNEVSIDRGMSSCLTNLECYCDNV------FLTSVQGDGLILSTTSGSTAYSLAAGG---SMVHPH
PpNADK2 DDNTSSESAEYLVLNEVSIDRGMSSALSNLECFCDGH------FVTIVQGDGLIISSPSGSTAYSLAAGG---SVVHPQ
PpNADK3 KEGDDPCEETHLVLNEIAIDRGMSSFLTNLECYCDDI------FLTSVQGDGLILSTPSGSTAYSLAAGG---SMVHPQ
PpNADK4 EGEDPCESEVHLVLNEVAIHRGMSSNLSNLECYCDGN------FVTSVQGDGLILSTPSGSTAYSLAAGG---SMVHPQ
CrNADK3 ------------VLNEVVIDRGISPFLTNLECYCDGS------LVTHVQGDGLIVATPTGSTAYNLAAGG---SMVHPQ
VcNADK3 ------------VLNEVVIDRGISSFLTNLECYCDGT------FVTHVQGDGLIVATPTGSTAYNLAAGG---SMVHPQ
PtNADK3 ------PGKVFDVLNEVVVDRGSNPYLSKIECYEHDR------LITKVQGDGVIVATPTGSTAYSTAAGG---SMVHPN
PtNADK2 ------PGKVFDVLNEVVVDRGSNPYLSKIECYEHDR------LITKVQGDGVIVATPTGSTAYSTSAGG---SMVHPN
BrNADK2 ------PGKVFDVLNEIVVDRGSNPYLSKIECYEHDR------LITKVQGDGVIVATPTGSTAYSTAAGG---SMVHPN
AtNADK2 ------PGKVFDVLNEIVVDRGSNPYLSKIECYEHDR------LITKVQGDGVIVATPTGSTAYSTAAGG---SMVHPN
VvNADK1 ------PGKIFDVMNEIVVDRGSNPYLSKIECYEHDR------LITKVQGDGVIVATPTGSTAYSTAAGG---SMVHPN
CsNADK2 ------PGKLFNILNEVVVDRGSNPYLSKIECYEHDR------LITKVQGDGVIVATPTGSTAYSTAAGG---SMVHPN
SiNADK3 ------PGKVFDVLNEVVVDRGSNPYLSKIECYEHDH------LITKVQGDGVIVATPTGSTAYSTAAGG---SMVHPN
OsNADK3 ------PGKVFDVLNEVVVDRGSNPYLSKIECYEHNH------LITKVQGDGVIVATPTGSTAYSTAAGG---SMVHPN
BdNADK3 ------PGKVFDVLNEVVVDRGSNPYLSKIECYEHNH------LITKVQGDGVIVATPTGSTAYSTAAGG---SMVHPN
SbNADK2 ------PGKVFDVLNEVVVDRGSNPYLSKIECYEHNH------LITKVQADGVIVATPTGSTAYSTAAGG---SMVHPN
ZmNADK1 ------PDKVFDVLNEVVVDRGSNPYLSKIECYEHNH------LITKVQGDGVIVATPTGSTAYSTAAGG---SMVHPN
SmNADK1 ------SGKVFEVLNEVVVDRGSNPYLCKIECYERNR------LITKVQADGVLVATPTGSTAYSTAAGG---SMVHPN
PpNADK1 ------PGKVFEVLNEVVVDRGSNPYLCKIECYERSR------LITKVQADGVIVATPTGSTAYSTAAGG---SMVHPN
CrNADK1 ------PEQVVEVLNEMVIDRGSSAFLTNIECYEKGR------FISRVQADGIMLATPTGSTAYSVAAGG---SMVHPN
VcNADK2 ------PEQSVEVLNEMVIDRGSSAFLTNIECYEKGR------FIARVQADGIMLATPTGSTAYSVAAGG---SMVHPN
CvNADK2(NC64A) AGWRAGCPEAFEVLNEVVLSRGANPYLSKIEVSEAGR------LITKVQADGVMLATPTGSTAYNVAAGG---SMVHPS
CsNADK2(C-169) ----NTPQELYEVLNEVVVDRGANPYLAKIECWERDT------LITKVQADGVMLATPTGSTAYSVAAGG---SMVHPN
MpNADK2(CCMP1545) -GGDGAPSHAYAVLNEVLVDRGPSPFLSKIEAYDRGL------FITTIQADGVMLATATGSTAYSVSAGG---SMVHPN
MpNADK1(RCC299) -GGGGTPSHTFTILNEVLVDRGPSPFLSKIEAYDRGQ------LITTIQADGVMLATATGSTAYSVSAGG---SMVHPN
OtNADK1 -GGTGQATKTVTVLNELLVDRGPSPYLSHIEAYDRGE------LITTIQADGVLVATATGSTAYSVSAGG---SMVHPN
OlNADK2 SGGTGEFTKKITVLNELLVDRGPSPYLSQIEAYDRGE------LITTIQADGVIVATATGSTAYSVSAGG---SMVHPN
GsNADK1 --------KEFQILNEVVVDRGASPYLCNLDCFCDNK------YITTVQADGIMSTPTGSTAYSMSAGG---SMVHPS
CmNADK2 --------QQFHVLNEVVVDRGASPFLCQLECYWDDA------PLASVQADGIIVASPTGSTAYSLAAGG---AMVHPS
GsNADK2 ASSKYKKVAQLHVLNDVVIDRGPAPFLSNLLCYCDEH------PVTRIQADGIIIATPTGSTAYSLSSGG---SMVHPA
CmNADK1 -----RPLIQKTVLNEVVVDRGPAPFLSNLECYCDDF------PVTRIQADGVILATPTGSTAYSLSSNG---SMVHPS
MpNADK1(CCMP1545) -------RRRHIVLNEVVIDRGANSTLIDLDVNIDGN------PMTKVLADGVMISTPTGSTAYSLAAGG---SMVHPG
MpNADK2(RCC299) -------RERHVVLNEIVVDRGARSQLIDLDVNVDGN------PMTKVLADGVMISTPTGSTAYALAAGG---SMVHPG
OlNADK1 --------QWRYVLNEVLIDRGPKPVMVELDIAVDGY------HVTKVAADGVILSSPTGSTAYSLAAGG---SMVHPG
OtNADK2 --------PWRYVLNEVLIDRGPKPVMVELDIAVDGY------KVTKVAADGVIVATATGSTAYSLAAGG---SMVHPG
VcNADK1 --------AVHHVLNECVLDRGAFPGAVLLEIFVDGS------YVTNVEADGLIISTPSGSTAYSMSAGG---PVVAPS
CrNADK2 --------EVHHVLNECVLDRGAFPGAVLLEIFIDGS------YVTNVEADGLIISTPSGSTAYSMSAGG---PVVAPS
CvNADK1(NC64A) --------RVHHVLNECLIDRGASPAMVQLECFVDGS------HITTAQADGLIIATPSGSTAYSMSAGG---PMVAPS
CsNADK1(C-169) --------AVHHVLNECLIDRGSSPSMVRLELYVDGH------HITTVRADGLIIATPSGSTAYSLSSGG---PMVAPS
CpNADK1 ---------MVHALNELAVHRGAELNMPEFQLFCGGR------LAATMPADGIIVSSATGSTAYALNAGG---PMVHPS
PtNADK5 ---------STYALNDILIADPCPATVSRFSFRIQRDSESCG-PLVNCRSSGLRVSTAAGSTAAMLSAGGFAMPVLSED
VvNADK3 ---------PTYALNDALLAHPCPATVSRCSFKIKREGHPCS-PLVHCRSSGLRVSTAAGSTAAMLSAGGFAMPILSQD
CsNADK3 ---------SKYPLNDVLIAHPCPASVSRFSFKIRNEQSCL---PLLNCRSSGLRVSTAAGSTAAMLSAGGFPMPILSQK
BrNADK6 ---------LSHALNDVLIAHPCPAAVSRFSFKIKSKDGYTSQ-KTVNCRSSGLRVCTAAGSTAAMLSAGGFVMPMLSRD
BrNADK5 ---------LSHALNDILIAHPCPAAVSKFSFKIKNKDCDTNP-KTVNCRSSGLRVCTAAGSTAAMLSAGGFLMPMLSRD
AtNADK3 ---------LSHALNDILIAQPCPAAVSRFSFKIKNKDGASSP-KTVNCRSSGLRICTAAGSTAAMQSAGGFVMPMLSRD
OsNADK4 ---------PTYALNDILVSHPCPASVSRFSFRKRSNTGESS-HLINCRSSGLRVSTATPAGSTAAMLSAGGFVMPISSHE
BdNADK4 ---------PTYALNDILVAHPCPASVSRFSLRKRSSTGETS-HLINSRSSGLRVSTATGSTAAMLSAGGFMMPISSRE
SiNADK4 ---------PTYALNDILVSHPCPASVSRFSFRKRSNTGEGS-HLINCRSSGLRVSTAAGSTAAMLSAGGFVMPLSSHE
ZmNADK4 ---------PTYALNDILVSHPCPASVSRFSFRKRNNTGENS-RLINCRSSGLRVSTAAGSTAAMLSAGGFTMPLSSRE
SbNADK4 ---------PTYALNDILVSHPCPASVSRFSFRKRNNMGETS-RLINCRSSGLRVSTAAGSTAAMLSAGGFMMPLSSRE
SmNADK4 ---------STPALNDVLLAHPNPAAVSRCTFSVVNQQTKGS-SLIHSRSSGLRVCTAAGSTAATLSAGGFAMPLESKE
PpNADK5 ---------DTPALNDILIAHPSPAAISRCSFSIEKQSTEELLIPVIHSRSSGLRISTATGSTAAMKSAGGTVMPLLSSK
MpNADK3(RCC299) ---------APALNDVLIAHPSPGAVSRYSVHVGGALGPP---LWFHVRSSGLRACTASGSTAAMRSAGGEPMHYLSRR
AmNADK1 --------WEALSLNEMLLHKEPLAGMCHFEIAVGEH------AVVDIASDGLLVSTPTGSTAYALAAGG---PVIAPG
PmNADK1 --------WESLCLNEMALHREPLTSMCHFEISIGRH------APVDISADGVILSTPTGSTAYSLSAGG---PVITPD
EcNADK1 --------RISTAINEVVLHPGKVAHMIEFEVYIDEI------FAFSQRSDGLIISTPTGSTAYSLSAGG---PILTPS
NE/D Motif
Gly-rich Motif

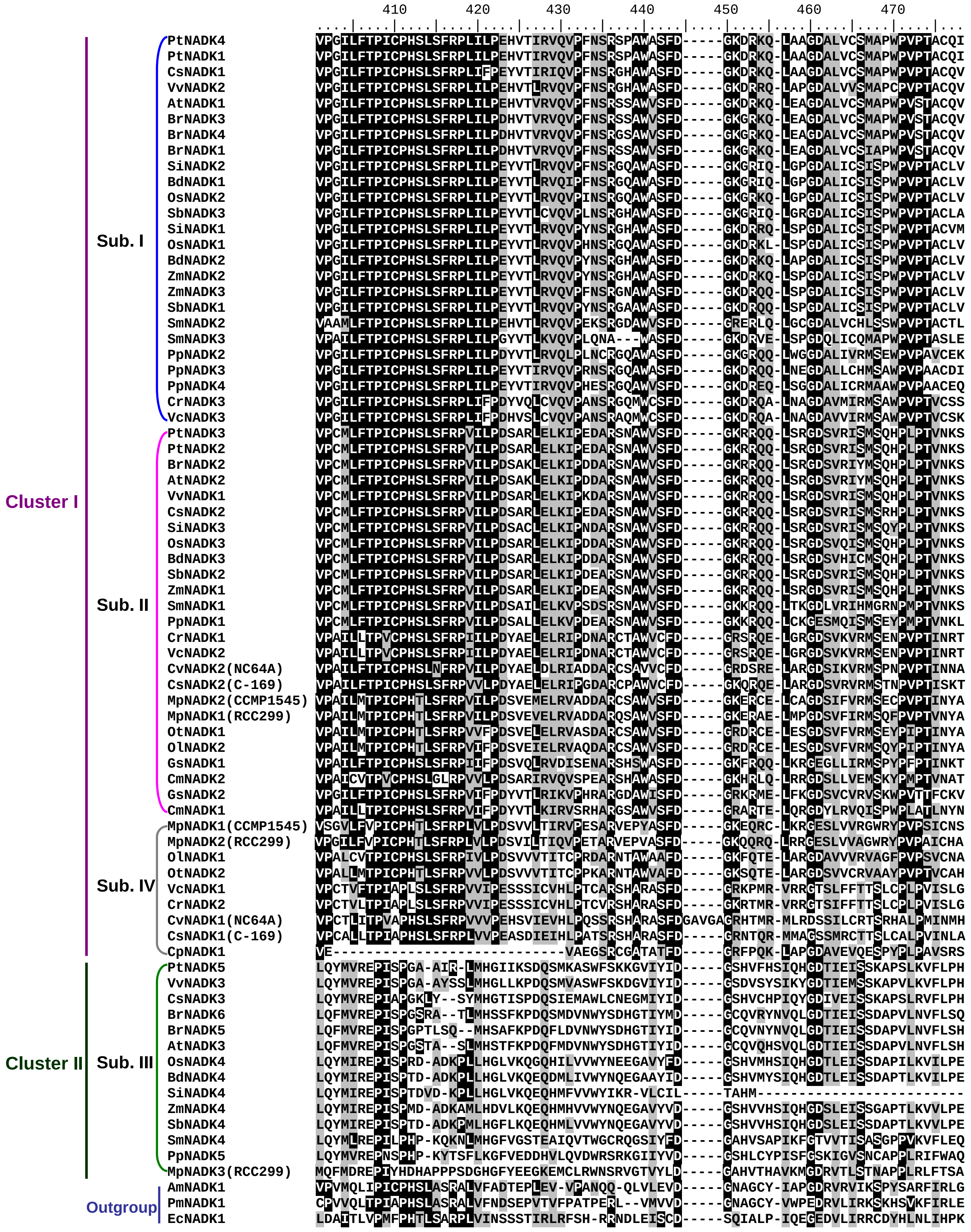
410 420 430 440 450 460 470
Cluster I
Sub. I
Sub. II
Sub. IV
Cluster II
Sub. III
Outgroup
PtNADK4 VPGILFTPICPHSLSFRPLILPEHVTIRVQVPFNSRSPAWASFD----GKDRKQ-LAAGDALVCSMAPWPVPTACQI
PtNADK1 VPGILFTPICPHSLSFRPLILPEHVTIRVQVPFNSRSPAWASFD----GKDRKQ-LAAGDALVCSMAPWPVPTACQI
CsNADK1 VPGILFTPICPHSLSFRPLIFPEYVTIRIQVPFNSRGHAWASFD----GKDRKQ-LAAGDALVCSMAPWPVPTACQV
VvNADK2 VPGILFTPICPHSLSFRPLILPEHVTLRVQVPFNSRGHAWASFD----GKDRRQ-LAPGDALVVSMAPCPVPTACQV
AtNADK1 VPGILFTPICPHSLSFRPLILPEHVTVRVQVPFNSRSSAWVSFD----GKDRKQ-LEAGDALVCSMAPWPVSTACQV
BrNADK3 VPGILFTPICPHSLSFRPLILPDHVTVRVQVPFNSRSSAWVSFD----GKGRKQ-LEAGDALVCSMAPWPVSTACQV
BrNADK4 VPGILFTPICPHSLSFRPLILPDHVTVRVQVPFNSRGSAWVSFD----GKGRKQ-LEAGDALVCSMAPWPVSTACQV
BrNADK1 VPGILFTPICPHSLSFRPLILPDHVTVRVQVPFNSRSSAWVSFD----GKGRKQ-LEAGDALVCSIAPWPVSTACQV
SiNADK2 VPGILFTPICPHSLSFRPLILPEYVTLRVQVPFNSRGQAWASFD----GKGRIQ-LGPGDALICSISPWPVPTACLV
BdNADK1 VPGILFTPICPHSLSFRPLILPEYVTLRVQIPFNSRGQAWASFD----GKGRIQ-LGPGDALICSISPWPVPTACLV
OsNADK2 VPGILFTPICPHSLSFRPLILPEYVTLRVQVPINSRGQAWASFD----GKGRKQ-LGPGDALICSISPWPVPTACLV
SbNADK3 VPGILFTPICPHSLSFRPLILPEYVTLCVQVPLNSRGHAWASFD----GKGRIQ-LGRGDALICSISPWPVPTACLA
SiNADK1 VPGILFTPICPHSLSFRPLILPEYVTLRVQVPYNSRGHAWASFD----GKDRRQ-LSPGDALICSISPWPVPTACVM
OsNADK1 VPGILFTPICPHSLSFRPLILPEYVTLRVQVPHNSRGQAWASFD----GKDRKL-LSPGDALICSISPWPVPTACLV
BdNADK2 VPGILFTPICPHSLSFRPLILPEYVTLRVQVPYNSRGHAWASFD----GKDRKQ-LAPGDALICSISPWPVPTACLV
ZmNADK2 VPGILFTPICPHSLSFRPLILPEYVTLRVQVPYNSRGHAWASFD----GKDRKQ-LSPGDALICSISPWPVPTACLV
ZmNADK3 VPGILFTPICPHSLSFRPLILPEYVTLRVQVPFNSRGNAWASFD----GKDRQQ-LSPGDALICSISPWPVPTACLV
SbNADK1 VPGILFTPICPHSLSFRPLILPEYVTLRVQVPYNSRGAAWASFD----GKDRQQ-LSPGDALICSISPWPVPTACLV
SmNADK2 VAAMLFTPICPHSLSFRPLILPEHVTLRVQVPEKSRGDAWVSFD----GRERLQ-LGCGDALVCHLSSWPVPTACTL
SmNADK3 VPAILFTPICPHSLSFRPLILPGYVTLKVQVPLQNA---WASFD----GKDRVE-LSPGDQLICQMAPWPVPTASLE
PpNADK2 VPGILFTPICPHSLSFRPLILPDYVTLRVQLPLNCRGQAWASFD----GKGRQQ-LWGGDALIVRMSEWPVPAVCEK
PpNADK3 VPGILFTPICPHSLSFRPLILPEYVTIRVQVPRNSRGQAWASFD----GKDRQQ-LNEGDALLCHMSAWPVPAACDI
PpNADK4 VPGILFTPICPHSLSFRPLILPEYVTIRVQVPHESRGQAWVSFD----GKDREQ-LSGGDALICRMAAWPVPAACEQ
CrNADK3 VPGILFTPICPHSLSFRPLIFPDYVQLCVQVPANSRGQMWCSFD----GKDRQA-LNAGDAVMIRMSAWPVPTVCSS
VcNADK3 VPGILFTPICPHSLSFRPLIFPDHVSLCVQVPANSRAQMWCSFD----GKDRQA-LNAGDAVVIRMSAWPVPTVCSK
PtNADK3 VPCMLFTPICPHSLSFRPVILPDSARLELKIPEDARSNAWVSFD----GKRRQQ-LSRGDSVRISMSQHPLPTVNKS
PtNADK2 VPCMLFTPICPHSLSFRPVILPDSARLELKIPEDARSNAWVSFD----GKRRQQ-LSRGDSVRISMSQHPLPTVNKS
BrNADK2 VPCMLFTPICPHSLSFRPVILPDSAKLELKIPDDARSNAWVSFD----GKRRQQ-LSRGDSVRIYMSQHPLPTVNKS
AtNADK2 VPCMLFTPICPHSLSFRPVILPDSAKLELKIPDDARSNAWVSFD----GKRRQQ-LSRGDSVRIYMSQHPLPTVNKS
VvNADK1 VPCMLFTPICPHSLSFRPVILPDSARLELKIPKDARSNAWVSFD----GKRRQQ-LSRGDSVRISMSQHPLPTVNKS
CsNADK2 VPCMLFTPICPHSLSFRPVILPDSARLELKIPEDARSNAWVSFD----GKRRQQ-LSRGDSVRISMSRHPLPTVNKS
SiNADK3 VPCMLFTPICPHSLSFRPVILPDSACLELKIPNDARSNAWVSFD----GKRRQQ-LSRGDSVRISMSQYPLPTVNKS
OsNADK3 VPCMLFTPICPHSLSFRPVILPDSARLELKIPDDARSNAWVSFD----GKRRQQ-LSRGDSVQISMSQHPLPTVNKS
BdNADK3 VPCMLFTPICPHSLSFRPVILPDSARLELKIPDDARSNAWVSFD----GKRRQQ-LSRGDSVHICMSQHPLPTVNKS
SbNADK2 VPCMLFTPICPHSLSFRPVILPDSARLELKIPDEARSNAWVSFD----GKRRQQ-LSRGDSVRISMSQHPLPTVNKS
ZmNADK1 VPCMLFTPICPHSLSFRPVILPDSARLELKIPDEARSNAWVSFD----GKRRQQ-LSRGDSVRISMSQHPLPTVNKS
SmNADK1 VPCMLFTPICPHSLSFRPVILPDSAILELKVPSDSRSNAWVSFD----GKKRQQ-LTKGDLVRIHMGRNPMPTVNKS
PpNADK1 VPCMLFTPICPHSLSFRPVILPDSALLELKVPDEARSNAWVSFD----GKKRQQ-LCKGESMQISMSEYPMPTVNKL
CrNADK1 VPAILLTPVCPHSLSFRPIILPDYAELELRIPDNARCTAWVCFD----GRSRQE-LGRGDSVKVRMSENPVPTINRT
VcNADK2 VPAILLTPVCPHSLSFRPIILPDYAELELRIPDNARCTAWVCFD----GRSRQE-LGRGDSVKVRMSENPVPTINRT
CvNADK2(NC64A) VPAILFTPICPHSLNFRPVILPDYAELDLRIADDARCSAVVCFD----GRDSRE-LARGDSIKVRMSPNPVPTINNA
CsNADK2(C-169) VPAILFTPICPHSLSFRPVVLPDYAELELRIPGDARCPAWVCFD----GKQRQE-LARGDSVRVRMSTNPVPTISKT
MpNADK2(CCMP1545) VPAILMTPICPHTLSFRPVILPDSVEMELRVADDARCSAWVSFD----GKERCE-LCAGDSIFVRMSECPVPTINYA
MpNADK1(RCC299) VPAILMTPICPHTLSFRPVILPDSVEVELRVADDARQSAWVSFD----GKERAE-LMPGDSVFIRMSQFPVPTVNYA
OtNADK1 VPAILMTPICPHTLSFRPVVFPDSVEELRVASDARCSAWVSFD----GRDRCE-LESGDSVFVRMSEYPIPTINYA
OlNADK2 VPAILMTPICPHTLSFRPVIFPDSVEIELRVAQDARCSAWVSFD----GRDRCE-LESGDSVFVRMSQYPIPTINYA
GsNADK1 VPAILFTPICPHSLSFRPIIFPDSVQLRVDISENARSHSWASFD----GKFRQQ-LKRGEGLLIRMSPYPFPTINKT
CmNADK2 VPAICVTPVCPHSLGLRPVVLPDSARIRVQVSPEARSHAWASFD----GKHRLQ-LRRGDSLLVEMSKYPMPTVNAT
GsNADK2 VPGILFTPICPHSLSFRPVIFPDYVTLRIKVPHRARGDAWISFD----GRKRME-LFKGDSVCVRVSKWPVTTFCKV
CmNADK1 VPAILLTPICPHSLSFRPVIFPDYVTLKIRVSRHARGSAWVSFD----GRARTE-LQRGDYLRVQISPWPLATLNYN
MpNADK1(CCMP1545) VSGVLFVPICPHTLSFRPLVLPDSVVLTIRVPESARVEPYASFD----GKEQRC-LKRGESLVVRGWRYPVPSICNS
MpNADK2(RCC299) VPGILFVPICPHTLSFRPLVLPDSVILTIQVPETARVEPVASFD----GKQQRQ-LRRGESLVVAGWRYPVPAICHA
OlNADK1 VPALCVTPICPHSLSFRPIVLPDSVVVTITCPRDARNTAWAAFD----GKFQTE-LARGDAVVVRVAGFPVPSVCNA
OtNADK2 VPALLMTPICPHTLSFRPVVLPDSVVVTITCPPKARNTAWVAFD----GKSQTE-LARGDSVVCRVAAYPVPTVCAH
VcNADK1 VPCTVFTPIAPLSLSFRPVVIPESSSICVHLPTCARSHARASFD----GRKPMR-VRRGTSLFFTTSLCPLPVISLG
CrNADK2 VPCTVLTPIAPLSLSFRPVVIPESSSICVHLPTCVRSHARASFD----GKRTMR-VRRGTSIFFTTSLCPLPVISLG
CvNADK1(NC64A) VPCTLITPVAPHSLSFRPVVVPEHSVIEVHLPQSSRSHARASFDGAVGAGRHTMR-MLRDSSILCRTSRHALPMINMH
CsNADK1(C-169) VPCALLTPIAPHSLSFRPLVVPEASDIEIHLPATSRSHARASFD----GRNTQR-MMAGSSMRCTTSLCALPVINLA
CpNADK1 VE-------------------------VAEGSRCGATATFD----GRFPQK-LAPGDAVEVQESPYPLPAVSRS
PtNADK5 LQYMVREPISPGA-AIR-LMHGIIKSDQSMKASWFSKKGVIYID----GSHVFHSIQHGDTIEISSKAPSLKVFLPH
VvNADK3 LQYMVREPISPGA-AYSSLMHGLLKPDQSMVASWFSKDGVIYID----GSDVSYSIKYGDTIEMSSKAPVLKVFLPH
CsNADK3 LQYMVREPIAPGKLY--SYMHGTISPDQSIEMAWLCNEGMIYID----GSHVCHPIQYGDIVEISSKAPSLRVFLPH
BrNADK6 LQFMVREPISPGSRA--TLMHSSFKPDQSMDVNWYSDHGTIYMD----GCQVRYNVQLGDTIEISSDAPVLNVFLSQ
BrNADK5 LQFMVREPISPGPTLSQ--MHSAFKPDQFLDVNWYSDHGTIYID----GCQVNYNVQLGDTIEISSDAPVLNVFLSH
AtNADK3 LQFMVREPISPGSTA--SLMHSTFKPDQFMDVNWYSDHGTIYID----GCQVQHSVQLGDTIEISSDAPVLNVFLSH
OsNADK4 LQYMIREPISPRD-ADKPLLHGLVKQGQHILVVWYNEEGAVYFD----GSHVMHSIQHGDTLEISSDAPILKVILPE
BdNADK4 LQYMIREPISPTD-ADKPLLHGLVKQEQDMLIVWYNQEGAAYID----GSHVMYSIQHGDTLEISSDAPTLKVILPE
SiNADK4 LQYMIREPISPTDVD-KPLLHGLVKQEQHMFVVWYIKR-VLCIL-----TAHM-----------------------
ZmNADK4 LQYMIREPISPMD-ADKAMLHDVLKQEQHMHVVWYNQEGAVYVD----GSHVVHSIQHGDSLEISSGAPTLKVVLPE
SbNADK4 LQYMIREPISPTD-ADKPMLHGFLKQEQHMLVVWYNQEGAVYVD----GSHVVHSIQHGDSLEISSDAPTLKVVLPE
SmNADK4 LQYMLREPILPHP-KQKNLMHGFVGSTEAIQVTWGCRQGSIYFD----GAHVSAPIKFGTVVTISASGPPVKVFLEQ
PpNADK5 LQYMVREPNSPHP-KYTSFLKGFVEDDHVLQVDWRSRKGIIYVD----GSHLCYPISFGSKIGVSNCAPPLRIFWAQ
MpNADK3(RCC299) MQFMDREPIYHDHAPPPSDGHGFYEEGKEMCLRWNSRVGTVYLD----GAHVTHAVKMGDRVTLSTNAPPLRLFTSA
AmNADK1 VPVMQLIPICPHSLASRALVFADTEPLEV-VPANQQ-QLVLEVD----GNAGCY-IAPGDRVRVIKSPYSARFIRLG
PmNADK1 CPVVQLTPIAPHSLASRALVFNDSEPVTVFPATPERL--VMVVD----GNAGCY-VWPEDRVLIRKSKHSVKFIRLE
EcNADK1 LDAITLVPMFPHTLSARPLVINSSSTIRLRFSH-RRNDLEISCD----SQIALP-IQEGEDVLIRRCDYHLNLIHPK

Figure S1. Amino acid sequences alignment analysis of NAD_Kinase domain of NADK proteins.

Amino acid sequences alignment and WebLogo analysis of NAD_Kinase domain in NADK genes from 24 species representing eight major lineages of the plantae: the glaucophytes (*Cyanophora paradoxa*), the rhodophytes (*Cyanidioschyzon merolae* and *Galdieria sulphuraria*), the chlorophytes (*Ostreococcus tauri*, *Ostreococcus lucimarinus*, *Micromonas pusilla* RCC299, *Micromonas pusilla* CCMP1545, *Chlorella variabilis* NC64A, *Coccomyxa subellipsoidea* C-169, *Chlamydomonas reinhardtii* and *Volvox carteri*), the bryophytes (*Physcomitrella patens*), the lycophytes (*Selaginella moellendorffii*), the gymnosperms (*Picea sitchensis*), the eudicots (*Arabidopsis thaliana, Populus trichocarpa*, *Cucumis sativus*, *Brassica rapa* and *Vitis vinifera*) and the monocots (*Brachypodium distachyon*, *Oryza sativa*, *Setaria italica*, *Sorghum bicolor* and *Zea mays*). Only CpNADK2 (ID, Contig53995) protein in *Cyanophora paradoxa* without complete NADK_Kinase domain is excluded, while the three outgroups of EcNADK1 (ID, EBESCP00000000630 or A7ZQ55) from *Escherichia coli* K-12, AmNADK1 (ID, AM1_1113 or B0C2T6) from *Acaryochloris marina* MBIC11017 and PmNADK1 (ID, P9301_14541 or A3PEA2) from *Prochlorococcus marinus* MIT 9301 are included in the alignment. Positions of the alignments where more than 50% of the sequences are identical are shown with black boxes. The four conserved NADK subfamilies are represented as sub. I, sub. II, sub, III and sub. IV at the left side of the alignment.
