# Supplementary material for: Genome-Wide Analysis of the NADK Gene Family in Plants
Source: PLoS One. 2014 Jun 26;9(6):e101051. doi: 10.1371/journal.pone.0101051 (PMC4072752; doi:10.1371/journal.pone.0101051)
Supplement: Figure S2 — Phylogenetic relationship and domain organization of NADK genes in plants, yeast and humans. (PDF) [file pone.0101051.s002.pdf]

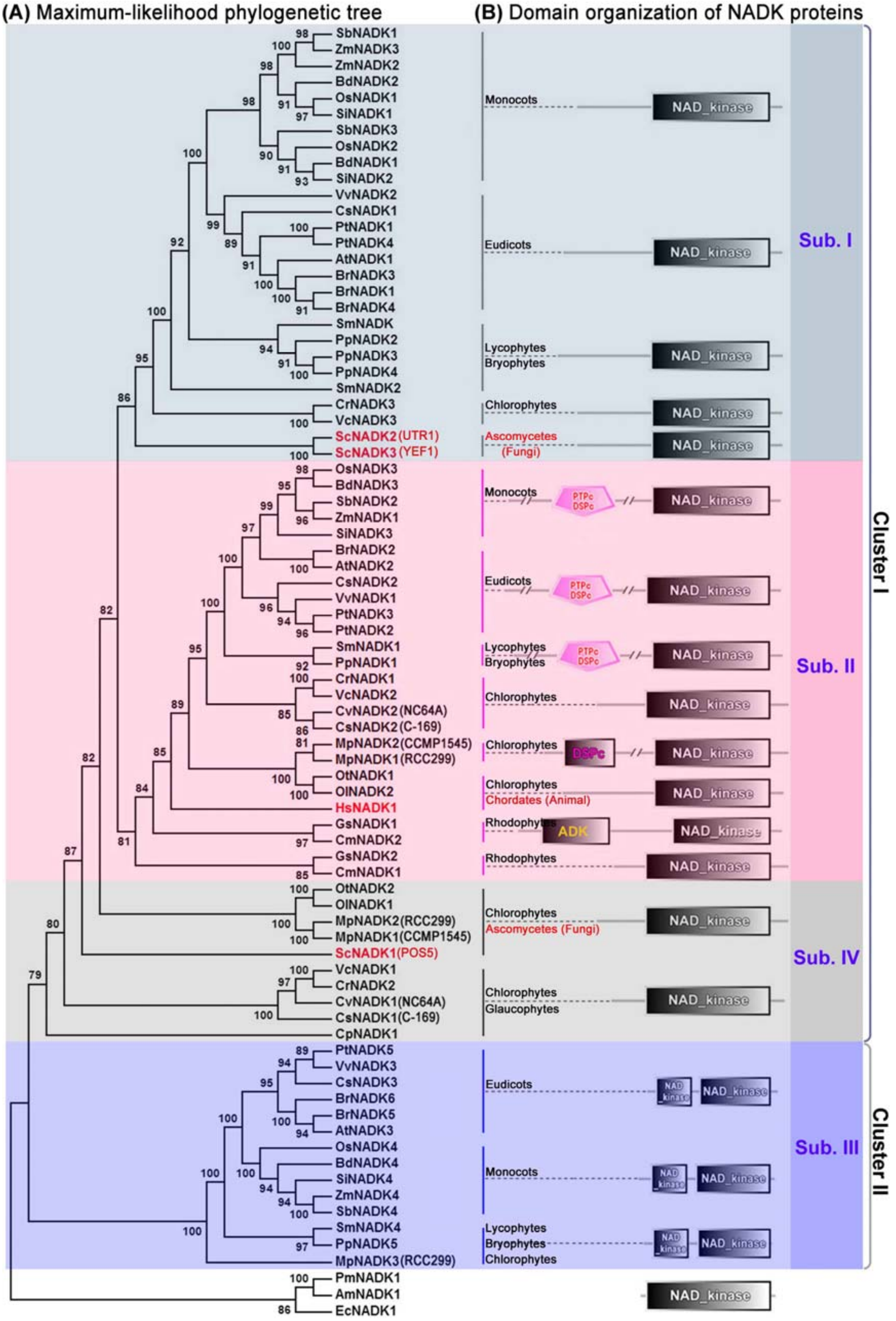

Figure S2. Phylogenetic relationship and domain organization of NADK genes in plants, yeast and humans.

(A) The rooted maximum-likelihood (ML) phylogenetic tree of NADK family was inferred from the amino acid sequences alignments of the NAD_kinase domain using the Phylip v3.68 package

under the gamma-corrected Jones-Taylor-Thornton (JTT) model. Bootstrap analyses involving 1,000 resamplings were performed. The NADK homologues from *Acaryochloris marina* MBIC11017, *Prochlorococcus marinus* MIT 9301 and *Escherichia coli* K-12 were selected as the out group. The results of Pfam and SMART analysis revealed that CpNADK2 has not complete NAD_kinase domain and was not included in ML tree. The Locus Name or IDs of ScNADK1~3 and HsNADK1 are YPL188W (POS5), YJR049C (UTR1), YEL041W (YEF1) and ENSG00000008130, respectively. **(B)** Domain organization of NADK proteins.
