# Supplementary material for: Genome-Wide Analysis of the NADK Gene Family in Plants
Source: PLoS One. 2014 Jun 26;9(6):e101051. doi: 10.1371/journal.pone.0101051 (PMC4072752; doi:10.1371/journal.pone.0101051)
Supplement: Figure S3 — Chromosomal locations of NADK family genes in plants. (PDF) [file pone.0101051.s003.pdf]

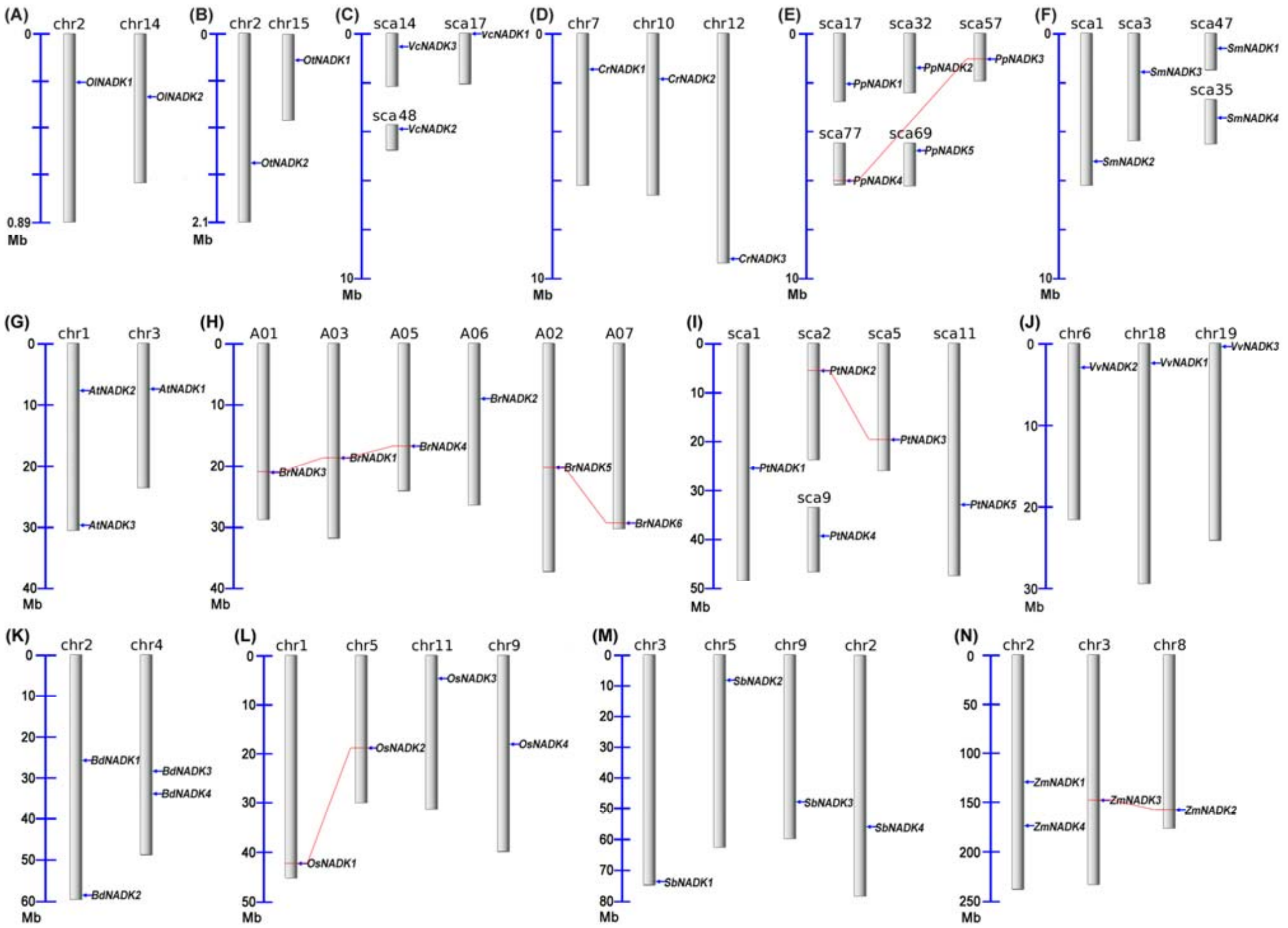


**Figure S3** Chromosomal locations of NADK family genes in plants.

Chromosomal locations of NADK family genes in algae *vir. Ostreococcus lucimarinus* **(A)**, *Ostreococcus tauri* **(B)**, *Volvox carteri* **(C)** and *Chlamydomonas reinhardtii* **(D)**; bryophytes *vir. Physcomitrella patens* **(E)**; lycophytes *vir. Selaginella moellendorffii* **(F)**; eudicots *vir. Arabidopsis thaliana* **(G)**, *Brassica rapa* **(H)**, *Populus trichocarpa* **(I)** and *Vitis vinifera* **(J)**; monocots *vir. Brachypodium distachyon* **(K)**, *Oryza sativa* **(L)**, *Sorghum bicolor* **(M)** and *Zea mays* **(N)**, respectively. The red lines connected two NADK genes stand for the duplication blocks of NADK homologues in intra-species (**Figure S4**).
