# Supplementary material for: Genome-Wide Analysis of the NADK Gene Family in Plants
Source: PLoS One. 2014 Jun 26;9(6):e101051. doi: 10.1371/journal.pone.0101051 (PMC4072752; doi:10.1371/journal.pone.0101051)
Supplement: Figure S4 — Segmental duplications and duplication and divergence times of NADK family genes in land plants. (PDF) [file pone.0101051.s004.pdf]

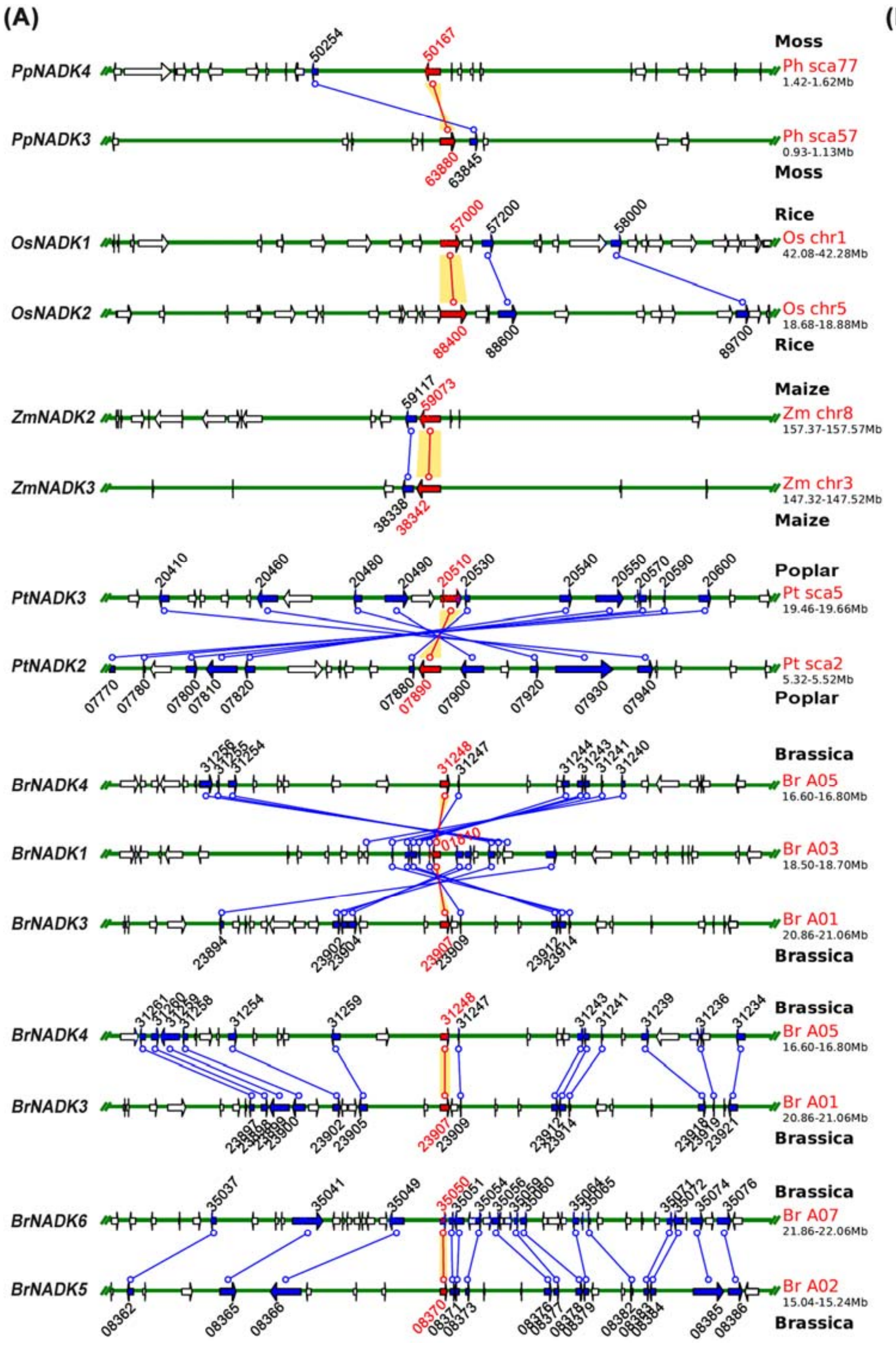

(B)

Ka	Ks	Ka/Ks	Duplication time (million years ago, Mya)
0.19	0.88	0.22	67.6
0.17	0.80	0.21	61.5
0.06	0.18	0.33	13.8
0.11	0.27	0.39	20.8
0.10	0.43	0.24	33.1
0.07	0.30	0.24	23.1
0.07	0.37	0.18	28.5

Figure S4 Segmental duplications and duplication and divergence times of NADK family genes in land plants. **(A)** Segmental duplications of NADK family genes in land plants. Paralogous gene pairs generated by segmental duplications in intra-species within NADK family genes were analyzed using the PGDD. All intra-species blocks for each query gene display regions of ±100 kb. Green lines connect gene pairs. Blue arrows indicate the other anchor homologous genes in the region, and red arrows indicate the query locus i.e NADK homologous genes. **(B)** The Ka, Ks, Ka/Ks ratios and estimated divergence time for each NADK paralogous gene pairs.
