# Supplementary material for: Genome-Wide Analysis of the NADK Gene Family in Plants
Source: PLoS One. 2014 Jun 26;9(6):e101051. doi: 10.1371/journal.pone.0101051 (PMC4072752; doi:10.1371/journal.pone.0101051)
Supplement: Figure S5 — Developmental expression patterns of NADK family genes in Arabidopsis and rice. (PDF) [file pone.0101051.s005.pdf]

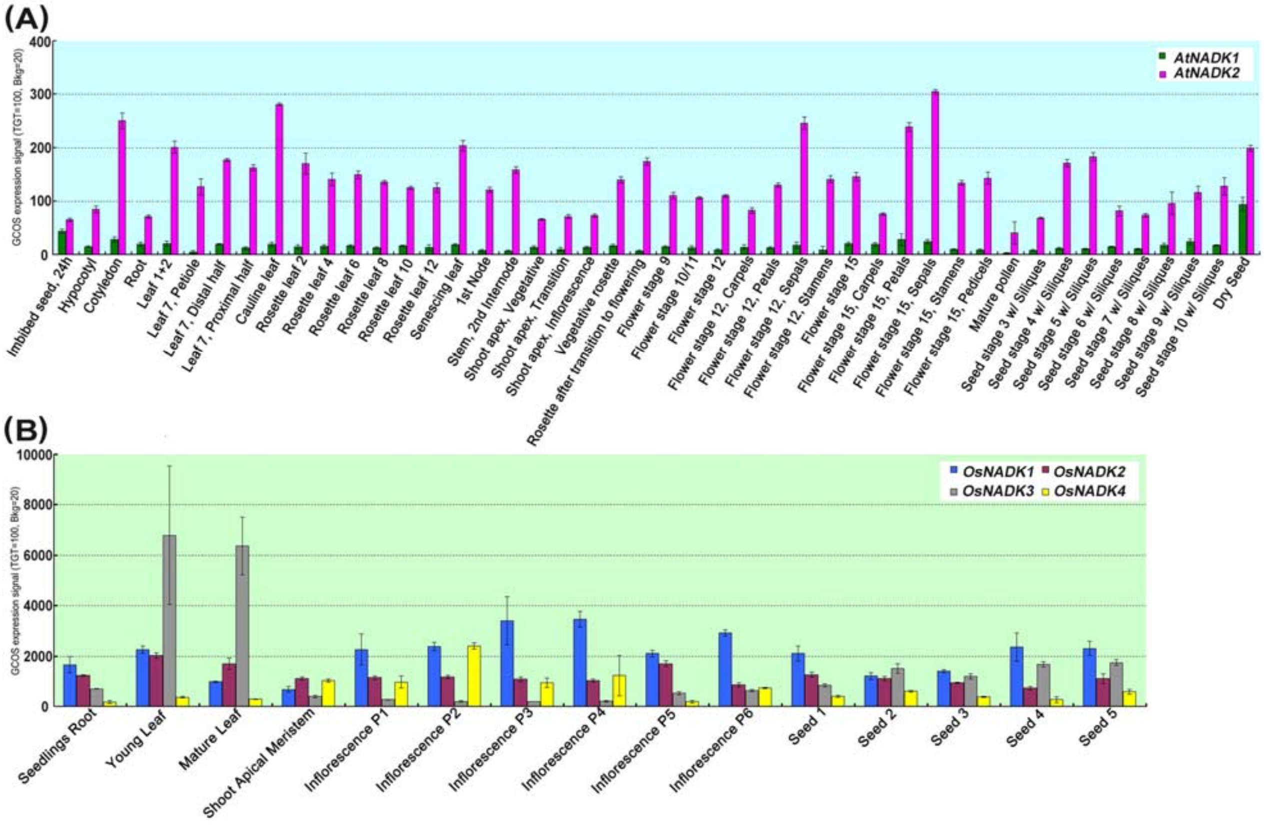

Figure S5 Developmental expression patterns of NADK family genes in Arabidopsis and rice.

Expression profiles obtained from (A) Arabidopsis and (B) rice microarray data in BAR (Bio-Analytic Resource, http://bar.utoronto.ca/welcome.htm), respectively. Results are shown as bar charts that reflect the absolutely expressive abundance of each NADK genes in different tissues. The *AtNADK3* was not available in BAR and its tissue expression patterns were not involved.
