# Supplementary material for: Genome-Wide Analysis of the NADK Gene Family in Plants
Source: PLoS One. 2014 Jun 26;9(6):e101051. doi: 10.1371/journal.pone.0101051 (PMC4072752; doi:10.1371/journal.pone.0101051)
Supplement: Figure S6 — Expression patterns of NADK family genes in Arabidopsis and rice under abiotic and biotic stresses. (PDF) [file pone.0101051.s006.pdf]

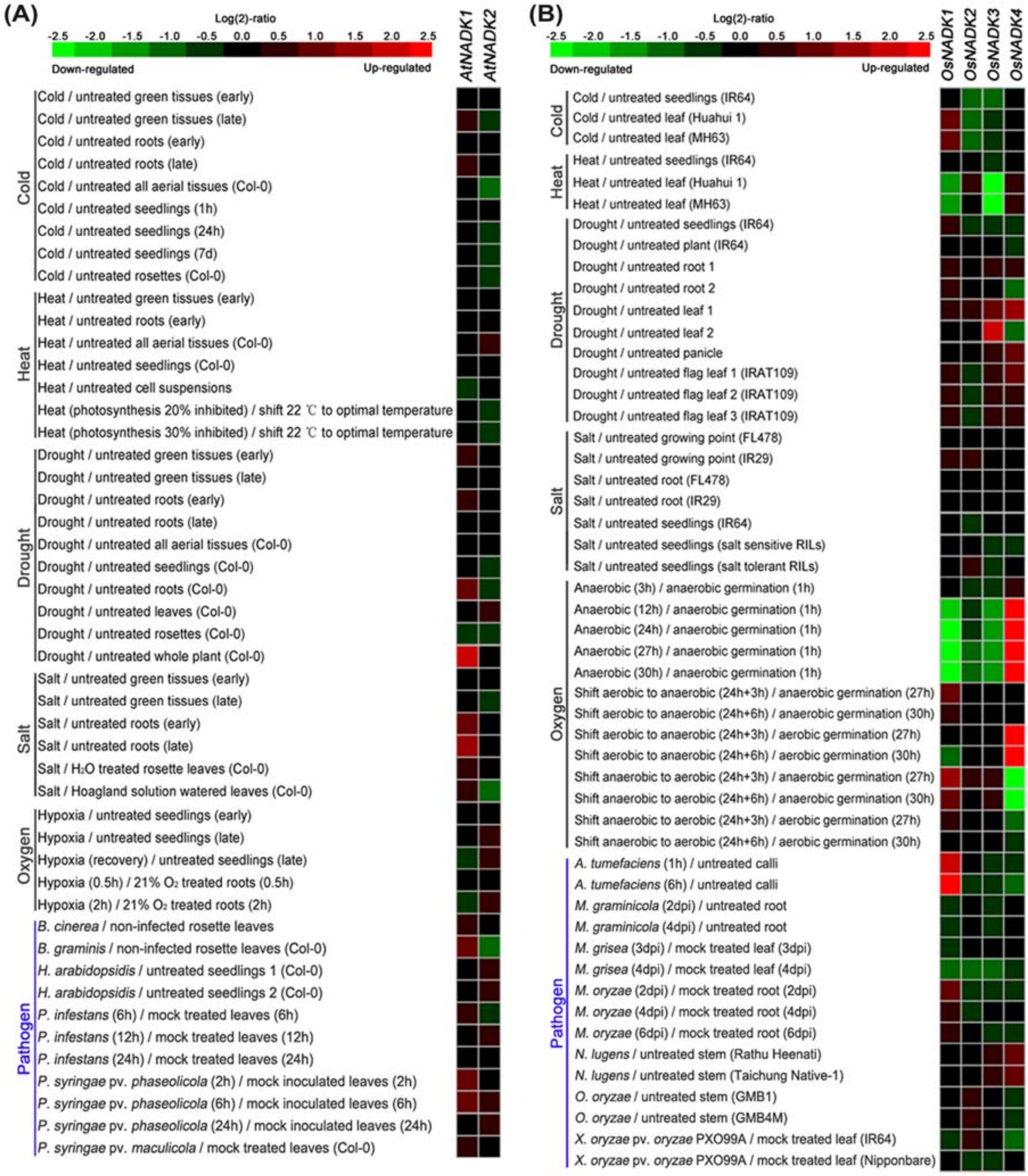

Figure S6 Expression patterns of NADK family genes in Arabidopsis and rice under abiotic and biotic stresses.

Expression profiles obtained from (**A**) Arabidopsis and (**B**) rice microarray data in Genevestigator illustrating differential expression of At*NADK1/2* and Os*NADK1–4* under different abiotic/biotic stresses, including cold, heat, drought, salt, hypoxia and pathogens. Results are shown as heat maps with green/red representing the indicated relative signal values.
