# Supplementary material for: Genome-Wide Analysis of the NADK Gene Family in Plants
Source: PLoS One. 2014 Jun 26;9(6):e101051. doi: 10.1371/journal.pone.0101051 (PMC4072752; doi:10.1371/journal.pone.0101051)
Supplement: Figure S7 — Expression patterns of NADK family genes in Arabidopsis and rice with various hormone treatments. (PDF) [file pone.0101051.s007.pdf]

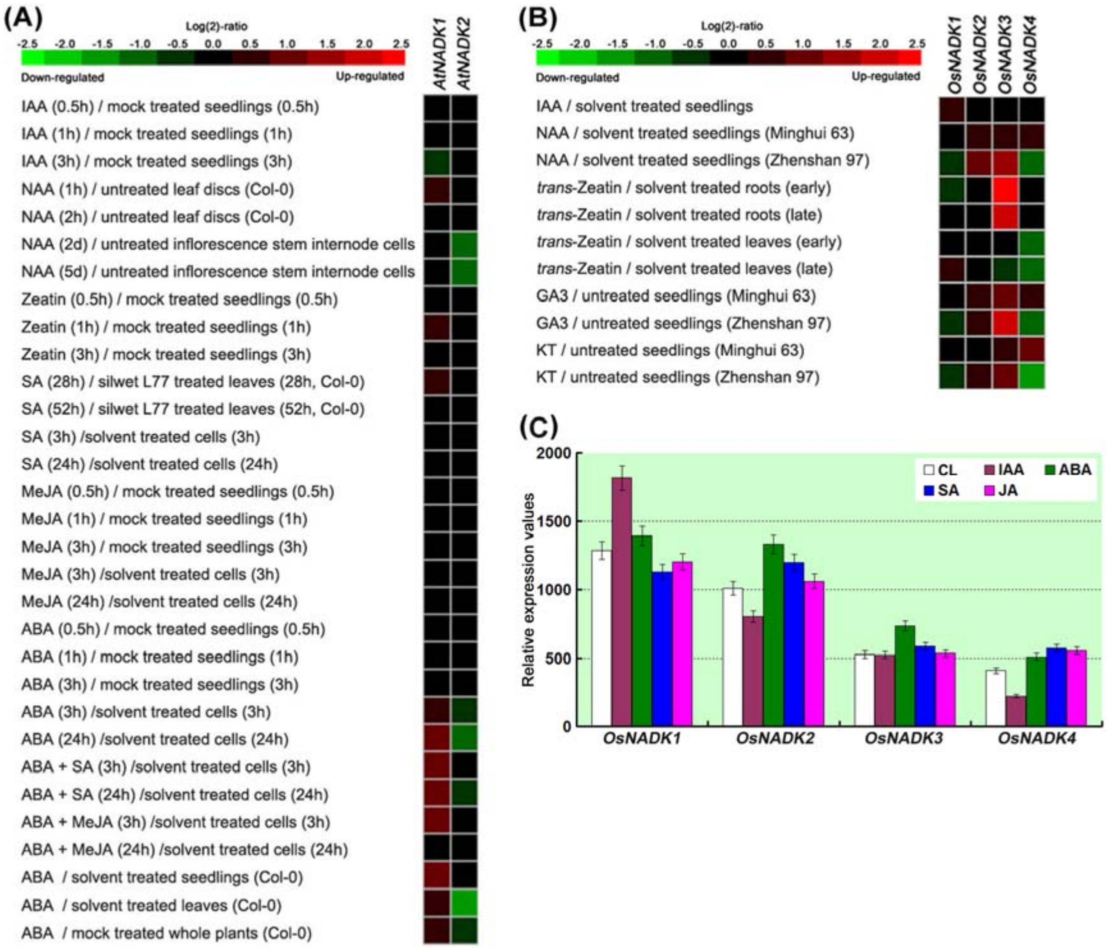

Figure 7 Expression patterns of NADK family genes in Arabidopsis and rice with various hormone treatments.

Expression profiles obtained from (**A**) Arabidopsis and (**B**) rice microarray data in Genevestigator illustrating differential expression of At*NADK1/2* and Os*NADK1–4* under different hormone treatments, including auxins (IAA, NAA), zeatin, SA, ABA, gibberellin and kinetin. Results are shown as heat maps with green/red representing the indicated relative signal values. (**C**) The relative expression of OsNADK genes in response to the hormones IAA, ABA, SA and JA compared to the control (CL), obtained from microarray (Gene Expression Omnibus, GEO; GSE37557). Values are the mean ± the standard deviation of three independent biological replicates.
