# Supplementary material for: Genome-Wide Analysis of the NADK Gene Family in Plants
Source: PLoS One. 2014 Jun 26;9(6):e101051. doi: 10.1371/journal.pone.0101051 (PMC4072752; doi:10.1371/journal.pone.0101051)
Supplement: Table S1 — The NADK family genes in plants. (PDF) [file pone.0101051.s008.pdf]

Table S1. The NADK family genes in plants

Lineages	Species*	Locus Name / ID[&]	Nomenclature	A. A.	Accession No.[#]	E value[$]	Subfamily
Bacteria	*Escherichia coli* K-12	EBESCP00000000630 (A7ZQ55)	*EcNADK1*	292	NP_417105.1	5.3E-110	Outgroup
Cyanobactes	*Acaryochloris marina* MBIC11017	AM1_1113 (B0C2T6)	*AmNADK1*	305	YP_001515466.1	2.6E-72	
	Prochlorococcus marinus MIT 9301	P9301_14541 (A3PEA2)	*PmNADK1*	302	YP_001091678.1	7.7E-79	
Glaucophytes	*Cyanophora paradoxa*	Contig7106	*CpNADK1*	280	NA	1.4E-15	Sub. IV
		Contig53995	*CpNADK2*	240	NA	6.1E-10	Excluded
Rhodophytes	*Cyanidioschyzon merolae*	CMP315C	*CmNADK1*	511	BAM81909.1	2.4E-57	Sub. II
		CMT143C	*CmNADK2*	877	BAM83146.1	4.2E-56	Sub. II
	Galdieria sulphuraria	452825095 (EME32094.1)	*GsNADK1*	738	EME32094.1	8.1E-57	Sub.II
		452825589 (EME32585.1)	*GsNADK2*	481	EME32585.1	6.5E-50	Sub.II
Chlorophytes	*Ostreococcus tauri*	4002 (gw1.15.00.172.1)	*OtNADK1*	721	XP_003083231.1	9.8E-57	Sub.II
		8188 (fgenesh1_pm.C_Chr_02.0001000063)	*OtNADK2*	874	XP_003075197.1	1.1E-51	Sub. IV
	Ostreococcus lucimarinus	35817 (e_gwEuk.2.225.1)	*OlNADK1*	314	XP_001416344.1	1.1E-54	Sub. IV
		43339 (e_gwEuk.14.189.1)	*OlNADK2*	313	XP_001421435.1	9.1E-50	Sub.II
	Micromonas pusilla RCC299	64658 (EuGene.1600010019)	*MpNADK1*(RCC299)	988	XP_002506643.1	2.7E-55	Sub. II
		67699 (gw2.05.171.1)	*MpNADK2*(RCC299)	311	XP_002502052.1	3.2E-56	Sub. IV
		84626 (e_gw2.08.331.1)	*MpNADK3*(RCC299)	309	XP_002507922.1	5.3E-06	Sub. III
	Micromonas pusilla CCMP1545	123035 (fgenesh2_kg.3_#_166_#_4275591:1)	*MpNADK1*(CCMP1545)	393	XP_003057482.1	3.2E-59	Sub. IV
		60995 (MicpuC2.EuGene.0000090546)	*MpNADK2*(CCMP1545)	836	XP_003061343.1	5.5E-50	Sub. II
	Chlorella variabilis NC64A	136701 (IGS.gm_17_00105)	*CvNADK1*(NC64A)	445	EFN53556.1	2.8E-51	Sub. IV
		143672 (IGS.gm_6_00246)	*CvNADK2*(NC64A)	499	EFN57008.1	2.7E-37	Sub. II
	Coccomyxa subellipsoidea C-169	49005 (estExt_fgenesh1_pg.C_200064)	*CsNADK1*(C-169)	342	EIE19085.1	6.6E-56	Sub. IV
		63074 (Genemark1.3857_g)	*CsNADK2*(C-169)	409	EIE23545.1	1.1E-59	Sub. II
	Chlamydomonas reinhardtii	Cre07.g322950.t1.2	*CrNADK1*	486	XP_001700866.1	1.2E-44	Sub. II
		Cre10.g431650.t1.2	*CrNADK2*	391	XP_001702496.1	4.9E-59	Sub. IV
		Cre12.g560600.t1.3	*CrNADK3*	355	XP_001703150.1	1.7E-39	Sub. I
	Volvox carteri	Vocar20000710m	*VcNADK1*	629	XP_002950024.1	1.1E-59	Sub. IV
		Vocar20012749m	*VcNADK2*	454	XP_002955100.1	1.2E-44	Sub. II
		Vocar20008225m	*VcNADK3*	354	XP_002949486.1	1.8E-33	Sub. I

Table S1. The NADK family genes in plants (Cont.)

Lineages	Species*	Locus Name / ID[&]	Nomenclature	A. A.	Accession No.[#]	E value[$]	Subfamily
Bryophytes	*Physcomitrella patens*	Pp1s17_312V6.1	*PpNADK1*	867	XP_001755443.1	5.9E-54	Sub. II
		Pp1s32_187V6.1	*PpNADK2*	582	XP_001758345.1	1.2E-56	Sub. I
		Pp1s57_138V6.1	*PpNADK3*	536	XP_001762814.1	1.2E-57	Sub. I
		Pp1s77_287V6.1	*PpNADK4*	589	XP_001765616.1	3.0E-61	Sub. I
		Pp1s69_47V6.1	*PpNADK5*	322	XP_001764605.1	3.3E-07	Sub. III
Lycophytes	*Selaginella moellendorffii*	50862 (gw1.47.338.1)	*SmNADK1*	714	XP_002981035.1	5.1E-58	Sub. II
		76799 (e_gw1.1.1339.1)	*SmNADK2*	378	XP_002962223.1	7.7E-58	Sub. I
		80525 (e_gw1.3.675.1)	*SmNADK3*	345	XP_002963632.1	1.2E-61	Sub. I
		107884 (e_gw1.35.564.1)	*SmNADK4*	312	XP_002977859.1	9.8E-06	Sub. III
Gymnosperms	*Picea sitchensis*	NA	NA	NA	NA	NA	NA
Eudicots	*Arabidopsis thaliana*	AT3G21070.1	*AtNADK1*	530	NP_974347.1	1.5E-52	Sub. I
		AT1G21640.2	*AtNADK2*	999	NP_001185057.1	5.1E-59	Sub. II
		AT1G78590.1	*AtNADK3*	317	NP_177980.2	2.6E-06	Sub. III
	Populus trichocarpa	Ptri.001G256100.1	*PtNADK1*	546	XP_002298393.1	7.9E-53	Sub. I
		Ptri.002G078700.1	*PtNADK2*	963	XP_002302220.1	2.4E-58	Sub. II
		Ptri.005G182600.1	*PtNADK3*	938	XP_002306654.1	2.5E-58	Sub. II
		Ptri.009G051500.1	*PtNADK4*	532	XP_002314082.1	1.2E-53	Sub. I
		Ptri.011G104400.1	*PtNADK5*	309	XP_002331318.1	2.4E-06	Sub. III
	Cucumis sativus	Cucsa.143470.1	*CsNADK1*	521	XP_004139039.1	9.9E-50	Sub. I
		Cucsa.328150.1	*CsNADK2*	984	XP_004149440.1	2.6E-57	Sub. II
		Cucsa.273630.1	*CsNADK3*	326	XP_004137227.1	1.5E-06	Sub. III
	Brassica rapa	Bra001810	*BrNADK1*	542	NA	1.3E-56	Sub. I
		Bra017934	*BrNADK2*	970	NA	2.7E-57	Sub. II
		Bra023907	*BrNADK3*	523	NA	3.9E-56	Sub. I
		Bra031248	*BrNADK4*	523	NA	1.9E-54	Sub. I
		Bra008370	*BrNADK5*	317	NA	1.8E-05	Sub. III
		Bra035050	*BrNADK6*	317	NA	1.6E-06	Sub. III

Table S1. The NADK family genes in plants (Cont.)

Lineages	Species*	Locus Name / ID[&]	Nomenclature	A. A.	Accession No.[#]	E value[$]	Subfamily
Eudicots	*Vitis vinifera*	GSVIVT01008810001	*VvNADK1*	846	XP_002284607.2	5.3E-56	Sub. II
		GSVIVT01025236001	*VvNADK2*	563	XP_002285357.1	3.9E-50	Sub. I
		GSVIVT01014095001	*VvNADK3*	315	XP_002283669.1	4.2E-07	Sub. III
Monocots	*Brachypodium distachyon*	Bradi2g26930.1	*BdNADK1*	548	XP_003568558.1	2.0E-60	Sub. I
		Bradi2g61240.1	*BdNADK2*	531	XP_003567496.1	5.0E-59	Sub. I
		Bradi4g23450.1	*BdNADK3*	973	XP_003577753.1	1.0E-55	Sub. II
		Bradi4g28380.1	*BdNADK4*	327	XP_003576439.1	1.0E-06	Sub. III
	Oryza sativa	LOC_Os01g72690.1	*OsNADK1*	571	NP_001045444.2	1.0E-58	Sub. I
		LOC_Os05g32210.1	*OsNADK2*	494	EEC79147.1	2.3E-62	Sub. I
		LOC_Os11g08670.1	*OsNADK3*	981	NP_001067415.1	6.9E-56	Sub. II
		LOC_Os09g17680.1	*OsNADK4*	325	NP_001062937.1	7.2E-07	Sub. III
	Setaria italica	Si000598m	*SiNADK1*	656	XP_004971313.1	1.3E-60	Sub. I
		Si021731m	*SiNADK2*	529	XP_004962206.1	2.3E-58	Sub. I
		Si025905m	*SiNADK3*	979	XP_004978897.1	2.9E-57	Sub. II
		Si030683m	*SiNADK4*	294	XP_004956632.1	6.3E-05	Sub. III
	Sorghum bicolor	Sb03g046360.1	*SbNADK1*	462	XP_002459132.1	5.8E-61	Sub. I
		Sb05g005750.1	*SbNADK2*	976	XP_002449145.1	1.7E-53	Sub. II
		Sb09g019130.1	*SbNADK3*	498	XP_002439730.1	1.7E-53	Sub. I
		Sb02g022560.1	*SbNADK4*	330	XP_002462255.1	7.4E-06	Sub. III
	Zea mays	GRMZM2G046498_T02	*ZmNADK1*	975	DAA38923.1	2.5E-51	Sub. II
		GRMZM2G059073_T02	*ZmNADK2*	569	NP_001147993.1	2.4E-58	Sub. I
		GRMZM2G138342_T01	*ZmNADK3*	565	NP_001151954.1	1.2E-60	Sub. I
		GRMZM2G006678_T01	*ZmNADK4*	331	ACF81218.1	8.6E-06	Sub. III

*, The genome data of above species are available, except for *Picea sitchensis* and *Galdieria sulphuraria*, and downloaded from the public database. The data sources of each species are following:

Bacteria: *Escherichia coli* K-12 (EcoGene v3.0, http://www.ecogene.org/)

Cyanobactes: *Acaryochloris marina* MBIC11017 (JGI, http://genome.jgi-psf.org/Acama_641228474/Acama_641228474.info.html)

Prochlorococcus marinus MIT 9301 (JGI, http://genome.jgi-psf.org/Proma_640069322/Proma_640069322.info.html)

Glaucophytes: *Cyanophora paradoxa* (http://cyanophora.rutgers.edu/cyanophora/home.php)

Rhodophytes: *Cyanidioschyzon merolae* (http://merolae.biol.s.u-tokyo.ac.jp/download/)
Galdieria sulphuraria (http://genomics.msu.edu/galdieria/)
Chlorophytes: *Ostreococcus tauri* (JGI v2.0, http://genome.jgi-psf.org/Ostta4/Ostta4.home.html)
Ostreococcus lucimarinus (JGI v2.0, http://genome.jgi-psf.org/Ost9901_3/Ost9901_3.home.html)
Micromonas pusilla RCC299 (JGI v3.0, http://genome.jgi-psf.org/MicpuN3/MicpuN3.home.html)
Micromonas pusilla CCMP1545 (JGI v3.0, http://genome.jgi-psf.org/MicpuC3/MicpuC3.home.html)
Chlorella variabilis NC64A (JGI v1.0, http://genome.jgi-psf.org/ChlNC64A_1/ChlNC64A_1.home.html)
Coccomyxa subellipsoidea C-169 (JGI v2.0, http://genome.jgi-psf.org/Coc_C169_1/Coc_C169_1.home.html)
Chlamydomonas reinhardtii (JGI v4.0, http://genome.jgi-psf.org/Chlre4/Chlre4.info.html)
Volvox carteri (JGI v2.0, http://genome.jgi-psf.org/Volca1/Volca1.info.html)
Bryophytes: *Physcomitrella patens* (JGI v3.0, http://genome.jgi-psf.org/Phypa1_1/Phypa1_1.home.html)
Lycophytes: *Selaginella moellendorffii* (JGI v1.1, http://genome.jgi-psf.org/Selmo1/Selmo1.home.html)
Gymnosperms: *Picea sitchensis* (NCBI, http://www.ncbi.nlm.nih.gov/)
Monocots: *Sorghum bicolor* (JGI v2.1, http://genome.jgi-psf.org/Sorbi1/Sorbi1.home.html)
Oryza sativa (RGAP v7.0, http://rice.plantbiology.msu.edu/index.shtml)
Zea mays (MaizeSequence v5.6, http://www.maizesequence.org/index.html)
Brachypodium distachyon (JGI v1.2, http://www.phytozome.net/brachy.php)
Setaria italica (JGI v2.1, http://www.phytozome.net/foxtailmillet.php)
Eudicots: *Arabidopsis thaliana* (TAIR v10.0, http://www.arabidopsis.org/)
Populus trichocarpa (JGI v2.2, http://genome.jgi-psf.org/Poptr1_1/Poptr2_2.home.html)
Vitis vinifera (GenoScope v1.0, http://www.genoscope.cns.fr/externe/GenomeBrowser/Vitis/)
Cucumis sativus (JGI v1.0, http://www.phytozome.net/cucumber.php)
Brassica rapa (BRAD v1.5, http://brassicadb.org/brad/)

[&], The original Locus Name or IDs are annotated in the database of each species.
[#], The corresponding Accession No. of each NADK is annotated in NCBI, and NA is not available.
[$], The E value of each NADK in hmmsearch by HMMER v3.0.
