# Supplementary material for: Genome-Wide Analysis of the NADK Gene Family in Plants
Source: PLoS One. 2014 Jun 26;9(6):e101051. doi: 10.1371/journal.pone.0101051 (PMC4072752; doi:10.1371/journal.pone.0101051)
Supplement: Table S2 — Cis -element analysis of the AtNADK and OsNADK promoters. (PDF) [file pone.0101051.s009.pdf]

Table S2 The *cis*-elements analysis of *AtNADKs* and *OsNADKs* promoters*

cis-elements	Core sequences	Experiments#	Functions of the *cis*-elements	*OsNADK1*	*OsNADK2*	*OsNADK3*	*OsNADK4*	*AtNADK1*	*AtNADK2*	*AtNADK3*
**Abiotic- / biotic-stress response elements**										
LTR	CCGAAA	Yes	*cis*-acting element involved in low-temperature responsiveness		+1285		+581			
HSE	AAAAAATTTC	No	*cis*-acting element involved in heat stress responsiveness	+1148	-1287	+1350	+553	-1208		-456
MBS	TAACTG	Yes	MYB binding site involved in drought-inducibility	+357, +1120	-457, -1259	+319, +903	-944			+1375
ARE	TGGTTT	Yes	*cis*-acting element essential for the anaerobic induction	-721, -890	+258	+13	+660	+279	+987	-808
TC-rich repeats	ATTCTCTAAC	No	*cis*-acting element involved in defense and stress responsiveness		+1412	-506			+508, +1384	-173
W box	TTGACC	Yes	elicitation; wounding and pathogen responsievness			+188, +1237		+60		
Box S	AGCCACC	No	elicitation; wounding and pathogen responsievness	+433						
GCC box	AGCCGCC	Yes	elicitation; wounding and pathogen responsievness				-1005			
EIRE	TTCGACC	Yes	elicitor-responsive element						+46	
Box-W1	TTGACC	Yes	fungal elicitor responsive element			+188,+1237		+60		-9, -834
**Hormone response elements**										
AuxRR-core	GGTCCAT	Yes	*cis*-acting element involved in auxin responsiveness			+945				
TGA-element	AACGAC	No	auxin-responsive element					-134	+829	
GARE-motif	AAACAGA	No	gibberellin-responsive element		-1294			+183		
ABRE	CACGTG	Yes	*cis*-acting element involved in abscisic acid responsiveness	+1367, +1328	+1020	-513	+125,+1289,+435,+1342	+295	+893	+812
ERE	ATTTCAAA	Yes	ethylene-responsive element	+1153	+642, +1374	+1344		+53		
TCA-element	GAGAAGAATA	No	*cis*-acting element involved in salicylic acid responsiveness					+555		+1151
CGTCA-motif	CGTCA	Yes	*cis*-acting element involved in MeJA-responsiveness	+62, +1373			+977, +1399	+310	+289	+156, +539

*, the *cis*-elements were scanned and identified by PlantCARE program (http://bioinformatics.psb.ugent.be/webtools/plantcare/html/) with the 1500 bp upstream from the transcription start site of each NADK gene. The numbers mean the positions of *cis*-elements at the promoter region, while the "+" and "-" stand for the positive and negative strands of the promoter sequences, respectively.

#, "Yes" means the *cis*-elements were identified on the basis of experimental evidence, while "No" means the *cis*-elements were putative and without relevant experimental evidence.
