# Supplementary material for: Genome-Wide Analysis of the NADK Gene Family in Plants
Source: PLoS One. 2014 Jun 26;9(6):e101051. doi: 10.1371/journal.pone.0101051 (PMC4072752; doi:10.1371/journal.pone.0101051)
Supplement: Table S3 — EST profiles of NADK genes in Arabidopsis . (PDF) [file pone.0101051.s010.pdf]

Table S3 The EST profiles of NADK family genes in Arabidopsis

Pool Name	Total ESTs	ESTs of		
		AtNADK1	*AtNADK2*	*AtNADK3*
**Root**	**49512**	AV547501.1	AV547169.1	AU226146.1<br>AU235446.1
**Leaf**	**12182**		BU636024.1<br>BU636736.1<br>CA781695.1	
**Vegetative tissue**	**2284**	BX835447.1<br>BX839123.1		
**Flower**	**62048**		ES108282.1	ES164751.1
**Silique**	**13014**			AV561000.1<br>AV566968.1<br>AV567142.1
**Seed**	**16148**		W43879.1	BE524882.1
**Other***		BP809334.1<br>BP832932.2<br>BP838976.1<br>BX836754.1<br>BX841206.1<br>DR255307.1<br>DR255308.1<br>DR255309.1<br>DR255310.1<br>DR255311.1<br>DR383743.1<br>EG510810.1<br>EG510811.1<br>EH860137.1<br>EL132995.1<br>EL190363.1	BP640307.1<br>BP821410.1<br>BX835190.1<br>BX838630.1<br>EH823802.1<br>EH871622.1<br>EH881615.1<br>EH922734.1<br>EH968762.1<br>EH991931.1<br>EL047134.1<br>EL122691.1<br>EL215449.1<br>AV799512.1<br>AV807413.1<br>AV813431.1<br>AV815254.1<br>AV822694.1<br>AV827816.1<br>BP562439.1<br>BP606150.1<br>BP617317.1<br>BP617652.1<br>BP862508.1	AV526752.1<br>BX837889.1<br>DR369581.1<br>DR369582.1<br>DR369583.1<br>DR369584.1<br>DR369585.1<br>DR369586.1<br>DR369587.1<br>DR369588.1<br>DR369589.1<br>DR384099.1<br>EL155433.1<br>EL327406.1<br>BP795858.1<br>BP613860.1<br>BP858918.1<br>BP866494.1
**Total**		19	30	25

*****, The cDNA library (EST pool) was not constructed by a single tissue, but by the mixed or whole plant or unspecified tissue, and the ESTs of which were excluded in the analysis of EST profiles (**Figure 5A**).
