# Supplementary material for: Genome-Wide Analysis of the NADK Gene Family in Plants
Source: PLoS One. 2014 Jun 26;9(6):e101051. doi: 10.1371/journal.pone.0101051 (PMC4072752; doi:10.1371/journal.pone.0101051)
Supplement: Table S4 — EST profiles of NADK genes in rice. (PDF) [file pone.0101051.s011.pdf]

Table S4 The EST profiles of NADK family genes in rice

Pool Name	Total ESTs	ESTs of			
		OsNADK1	***OsNADK2***	***OsNADK3***	***OsNADK4***
**Callus**	**162069**	CI309114.1	CF322589.1	CI369244.1	BP185052.1
		CI067920.1	CI378330.1	CI371157.1	CF320309.1
		CI310141.1	CF330661.1	CI386010.1	CF323256.1
		CI071661.1	CF330662.1	CI607792.1	CI374620.1
		CI078840.1			
		CI081084.1			
		CI083780.1			
		AU100967.1			
		C28575.1			
		CF306707.1			
		CF322444.1			
		CF322737.1			
		CI605789.1			
		CI375206.1			
		CI612152.1			
		CI378715.1			
		CI615150.1			
		CI381140.1			
		CI616362.1			
		CI384318.1			
		CI385295.1			
		CI619349.1			
		CI387387.1			
		CI621064.1			
		CI388476.1			
		CI621752.1			
		CI622304.1			
		CI390522.1			
		CI623608.1			
**Root**	**67194**	CA753682.1	CI538761.1	CI553381.1	
		CI757906.1	CI540594.1		
		CI541204.1	CI544776.1		
		CI547747.1	CA757976.1		
		CI764339.1			
		CI552904.1			
		CI769209.1			
		CI557176.1			
		CI773193.1			
**Leaf**		CB673330.1	CI422742.1	BI811834.1	
		CB673331.1	CI649510.1	BI812492.1	
		BM420996.1	CF326325.1	BI812533.1	
		CF954946.1	CB637120.1	BM421122.1	
			BM420922.1	CB640927.1	

Table S4 The EST profiles of NADK family genes in rice (Cont.).

Pool Name	Total ESTs	ESTs of			
		OsNADK1	*OsNADK2*	*OsNADK3*	*OsNADK4*
			CF952780.1	CB640936.1	
				CB641148.1	
				CB641149.1	
				CB668601.1	
				CB668602.1	
				CB672816.1	
				CB672817.1	
				CB621210.1	
**Leaf**	**175658**			CB621211.1	
				CB631182.1	
				CB631183.1	
				CB631712.1	
				CB631713.1	
				CB631745.1	
				CB631746.1	
				CB634918.1	
				CB634919.1	
		CI290106.1		CI000551.1	CI294744.1
		CI011570.1		CI002744.1	CI582348.1
		CI315462.1		CI003993.1	CI025692.1
		CI565240.1		CI285451.1	
		CI315995.1		CI312590.1	
		CI318870.1		CI313050.1	
		CI567931.1		CI313860.1	
		CI320801.1		CI314419.1	
		CI324799.1		CI314637.1	
		CI574277.1		CI314673.1	
		CI326861.1		CI315226.1	
		CI576072.1		CI315751.1	
		CI338448.1		CI316766.1	
**Stem**	**124773**	CI584393.1		CI318471.1	
				CI319487.1	
				CI319632.1	
				CI320490.1	
				CI325433.1	
				CI329066.1	
				CI330978.1	
				CI331056.1	
				CI331479.1	
				CI331858.1	
				CI332320.1	
				CI336101.1	
				CI338661.1	

Table S4 The EST profiles of NADK family genes in rice (Cont.).

**Pool Name**	**Total ESTs**	**ESTs of**			
		OsNADK1	***OsNADK2***	***OsNADK3***	***OsNADK4***
**Stem**				CI564123.1	
**Flower**	**134391**	CI339943.1<br>CI348716.1<br>CI592026.1<br>CI349851.1<br>CI595224.1<br>CI354242.1<br>CI357321.1<br>CI598268.1<br>CI360807.1<br>CI517541.1<br>CI735815.1<br>CI523464.1<br>CI741444.1	CI345121.1<br>CI356802.1<br>CI597776.1<br>CI597937.1<br>CI363229.1<br>CI364923.1	CI339367.1<br>CI347085.1<br>CI354175.1<br>CI518671.1<br>CI522801.1<br>CI526437.1<br>CI527302.1<br>CI529495.1<br>CI531006.1<br>CI535215.1	
**Panicle**	**136249**	CI106258.1<br>CI112020.1<br>CI112565.1<br>CI113175.1<br>CK044828.1<br>CK055927.1<br>CK061719.1<br>CK063004.1	EG709852.1<br>CA762987.1		
**Seed**	**32409**	CI736601.1<br>CI518864.1<br>CI737067.1	CI520833.1<br>CI396842.1		
**Other***		CI118744.1<br>CI223352.1<br>CI248486.1<br>CT859916.1<br>BX899993.1<br>FG952358.1	CI744756.1<br>CR287023.1<br>CX106324.1<br>CX110024.1<br>CI258913.1<br>CI225558.1	CI273468.1<br>EX449817.1<br>EX449818.1<br>CI472611.1<br>CX118627.1	CT858530.1<br>EX451307.1<br>CX104330.1
**Total**		86	30	70	10

*****, The cDNA library (EST pool) was not constructed by a single tissue, but by the mixed or whole plant or unspecified tissue, and the ESTs of which were excluded in the analysis of EST profiles (**Figure 5B**).
