# Supplementary material for: Genome-Wide Analysis of the NADK Gene Family in Plants
Source: PLoS One. 2014 Jun 26;9(6):e101051. doi: 10.1371/journal.pone.0101051 (PMC4072752; doi:10.1371/journal.pone.0101051)
Supplement: Table S5 — The gene-specific primers used for qRT-PCR in this study. (PDF) [file pone.0101051.s012.pdf]

Table S5 The gene-specific primers used for qRT-PCR in this study

Gene Name	Primer Sequences	Amplicon Length	Span Introns
AtNADK1	F: 5’-TGCTACTGTGACAACTCATTCGT-3’<br>R: 5’-TTGAATGGCACTTGGACTCTC-3’	218 bp	10
AtNADK2	F: 5’-GTTATGAGCACGACCGTCTTATC-3’<br>R: 5’-TGTGGGCAGATTGGAGTGAAC-3’	151 bp	5, 6
AtNADK3	F: 5’-TCAATTCTGACCCAACACAAGC-3’<br>R: 5’-AGGAACCACTCTGCCAAACAA-3’	146 bp	2, 3
AtTub6<br>(AT5G12250)	F: 5’-ATGTTGTACGCAAAGAGGCTG-3’<br>R: 5’-AAGTGTCGCATTGTATGGCTC-3’	209 bp	1
OsNADK1	F: 5’-GATGGGACTGTTTTATGGGCT-3’<br>R: 5’-CATGGCACTGTAGACGGTTTC-3’	181 bp	7, 8
OsNADK2	F: 5’-TGACTAAACCTAATTCCAACTCCG-3’<br>R: 5’-ACAATGATGCTGCCCATAAAAC-3’	246 bp	5, 6
OsNADK3	F: 5’-CCTTCCTGATTCTGCTCGTCTT-3’<br>R: 5’-CCTTGATAATTGCTGCCTCCTT-3’	100 bp	7
OsNADK4	F: 5’-TGCGTGTATCCAATCCCAAG-3’<br>R: 5’-TAGCCCGTAGAAGAGTGCCAT-3’	201 bp	1
OsActin1<br>(accession ID KC140126)	F: 5’-GTGGTCGCCCCTCCTGAAAG-3’<br>R: 5’-GGCTTAGCATTCTTGGGTCCG-3’	170 bp	3
